# Evolutionary classification of ammonium, nitrate, and peptide transporters in land plants

**DOI:** 10.1186/1471-2148-14-11

**Published:** 2014-01-20

**Authors:** Neil JJB von Wittgenstein, Cuong H Le, Barbara J Hawkins, Jürgen Ehlting

**Affiliations:** 1 Centre for Forest Biology & Department of Biology, University of Victoria, PO Box 1700 STN CSC, Victoria, BC V8W 2Y2, Canada; 2 Centre for Forest Biology & Department of Biochemistry and Microbiology, University of Victoria, PO Box 1700 STN CSC, Victoria, BC V8W 2Y2, Canada

**Keywords:** Ammonium transporter (AMT1 and AMT2), Nitrate transporter (NRT1 and NRT2), Peptide transporter (PTR), Gene family evolution

## Abstract

**Background:**

Nitrogen uptake, reallocation within the plant, and between subcellular compartments involves ammonium, nitrate and peptide transporters. Ammonium transporters are separated into two distinct families (AMT1 and AMT2), each comprised of five members on average in angiosperms. Nitrate transporters also form two discrete families (NRT1 and NRT2), with angiosperms having four NRT2s, on average. NRT1s share an evolutionary history with peptide transporters (PTRs). The NRT1/PTR family in land plants usually has more than 50 members and contains also members with distinct activities, such as glucosinolate and abscisic acid transport.

**Results:**

Phylogenetic reconstructions of each family across 20 land plant species with available genome sequences were supplemented with subcellular localization and transmembrane topology predictions. This revealed that both AMT families diverged prior to the separation of bryophytes and vascular plants forming two distinct clans, designated as supergroups, each. Ten supergroups were identified for the NRT1/PTR family. It is apparent that nitrate and peptide transport within the NRT1/PTR family is polyphyletic, that is, nitrate and/or peptide transport likely evolved multiple times within land plants. The NRT2 family separated into two distinct clans early in vascular plant evolution. Subsequent duplications occurring prior to the eudicot/monocot separation led to the existence of two AMT1, six AMT2, 31 NRT1/PTR, and two NRT2 clans, designated as groups.

**Conclusion:**

Phylogenetic separation of groups suggests functional divergence within the angiosperms for each family. Distinct groups within the NRT1/PTR family appear to separate peptide and nitrate transport activities as well as other activities contained within the family, for example nitrite transport. Conversely, distinct activities, such as abscisic acid and glucosinolate transport, appear to have recently evolved from nitrate transporters.

## Background

Nitrogen (N) is the macronutrient required by plants in the greatest amounts, yet N is often the most limiting nutrient in terrestrial ecosystems, due to its low availability in soils [1,2]. In soils, N can exist as organic N, in the forms of amino acids, free peptides, and proteins [3,4], and as inorganic N, in the forms of nitrate (NO_3_^-^) and ammonium (NH_4_^+^) [4]. Inorganic N is the most prominent form of N taken up by many land plant species [5,6]. NH_4_^+^ and NO_3_^-^ uptake from the soil, as well as movement of NH_4_^+^ and NO_3_^-^ throughout the plant, is regulated by current N demand for growth and storage, and is largely performed by two groups of ion transporter proteins, NH_4_^+^ transporters (AMTs) and NO_3_^-^ transporters (NRTs) [4,7]. Each group can be subdivided into two families based on sequence similarity: NRT1 and NRT2, and AMT1 and AMT2. NRT1’s are part of a large family of solute transporters that also includes peptide transporters (PTR).

NRTs are encoded by two distinct gene families (*NRT1* and *NRT2*) that do not share significant overall sequence similarity. Both families perform proton-coupled active transport and have 12 putative transmembrane (TM) domains [5]. The NRT2 family is responsible for the high affinity transport system (HATS) of NO_3_^-^[8]. The HATS is composed of saturable transporters that take up NO_3_^-^ at low rates and high affinity and are expressed under NO_3_^-^ limiting conditions. The HATS has inducible members (iHATS), which are expressed in response to low NO_3_^-^ concentrations, as well as constitutive members (cHATS), which are not N-inducible [9]. Some members of the NRT2 family require physical association (protein interaction) with NAR2 (Nitrate Assimilation-Related) proteins [10,11] for proper functioning. Interaction with NAR2 proteins was shown to be necessary in diverse plant lineages, including monocots, eudicots, and green algae [9,12].

NRT1s are responsible for the low affinity transport system (LATS) of NO_3_^-^. The LATS contains non-saturable transporters that transport NO_3_^-^ at much higher rates than the HATS and are expressed under NO_3_^-^ abundant conditions [8]. More than fifty putative members of the family have been identified in *A. thaliana*; however, many of these are not NO_3_^-^ transporters but more likely encode transporters of other N-containing compounds such as small peptides or amino acids [8]. Recently, NRT1/PTR family members have also been shown to transport solutes with distinct physiological functions, such as the plant hormone abscisic acid (ABA) [13] or herbivore-deterring glucosinolates [14].

Both AMT1s and AMT2s contain 11 putative TM domains [6,15,16]. The AMT1 family largely comprises members responsible for high affinity NH_4_^+^ transport [17]. AMT1s are channel-like proteins [18] that act as NH_4_^+^ uniporters or NH_3_/H^+^ cotransporters [19]. AMT1 and AMT2s do share a distant common evolutionary history and the superfamily includes the Rh family of ammonium transporters present in green algae, but not in land plants. AMT1s are more closely related to prokaryotic ammonium transporters than they are to AMT2s and were likely inherited vertically [16]. In contrast, plant AMT2s (referred to as MEPα in [16]) form a sister clan to some fungal proteins from leotiomyceta and several horizontal gene transfers are apparent in the larger MEP family [16]. In general, the physiological roles of AMT2 proteins are less well understood than those of AMT1 proteins [20,21]. The *Lotus japonicus LjAMT2-2* is involved in NH_3_ uptake through mycorrhizal symbiosis [22]. AMT2s do not exist in most green algae, but they are present in *Mamiellales*, although these AMT2s do not share a common evolutionary origin with land plant AMT2s. McDonald *et al.*[16] suggested that land plant AMT2s share a common origin with AMT2s from *Archaea*, while a separate horizontal gene transfer event from bacteria may have been responsible for the AMT2s in *Mamiellales*.

Here, we present comprehensive phylogenies reconstructing the evolutionary history of the NH_4_^+^, NO_3_^-^ and peptide transporter families, AMT1, AMT2, NRT1/PTR, and NRT2 across 20 fully sequenced land plant (*Embryophyta*) genomes complemented with two green algal (*Chlorophyta*) species. These phylogenies are supplemented with TM domain topology predictions, subcellular localization predictions, and *in silico* expression profiling for selected species. All four N transporter families appear to be monophyletic in plants. However, all four families in angiosperms contain members that separated early during land plant evolution and that further diverged through gene duplications prior to the monocot/eudicot split to give rise to evolutionarily and functionally distinct groups. This provides the basis to build hypotheses on physiological functions of NH_4_^+^, NO_3_^-^, and peptide transporters, and suggests a classification system for the transporter families based on their evolutionary relationships.

## Results and discussion

**Figure 1:**
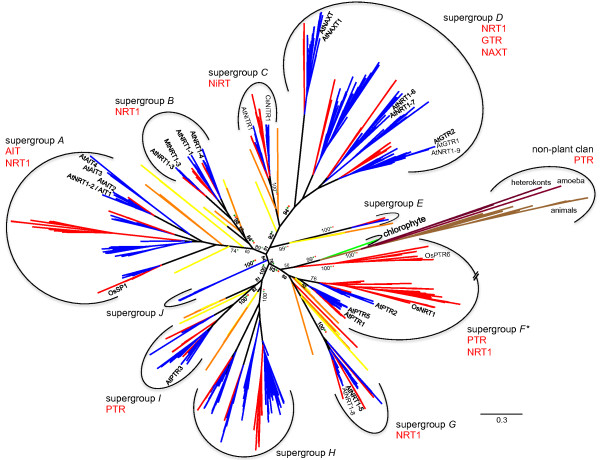
**NRT1/PTR phylogeny.** Unrooted maximum likelihood phylogenetic reconstruction of the NRT1/PTR families in plants and a set of 24 non-plant sequences identified as best GenBank BLAST hits using representative members from each supergroup as query. Taxonomic groups are colored such that blue refers to eudicots, red to monocots, green to chlorophytes, yellow to bryophytes, and orange to lycophytes. Percent Bootstrap values from 1,000 replicates are given for central branches only up to the branches defining supergroups. The approximate location of functionally characterized transporters discussed is indicated (NRT: nitrate transporter, GTR: glucosinolate transporter, PTR: peptide transporter, NiTR: nitrite (NO_2_^-^) transporter, NAXT: nitrate excretion transporter, and AIT: abscisic acid (ABA) transporter). *Note that supergroup F is paraphyletic; the containing clades have been combined owing to poor bootstrap support separating them. For detailed phylogenies of each superfamily and group definitions see Additional file 2. For database accession numbers of the 1,101 protein sequences included see Additional file 1.

**Figure 2:**
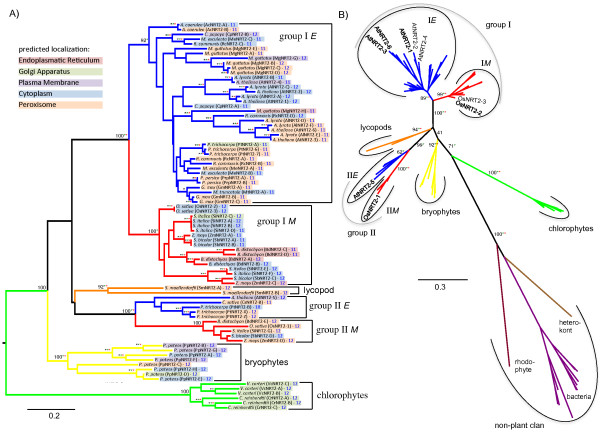
**Maximum Likelihood phylogenetic reconstruction of the NRT2 family.** Taxonomic groups are colored as described in Figure 1. **A)** Phylogram of the NRT2 family including 81 plant sequences only. Chlorophyte sequences were used to root the tree. Bootstrap values of the ML analysis are given and corresponding bootstrap values (>75%) from a distance neighbor joining and a parsimony analysis are indicated as green and red stars, respectively (within groups, bootstrap values >75% are displayed as stars only for all three analyses). Subcellular localization predictions are indicated as colored boxes framing gene identifiers and transmembrane topology predictions are given as numbers to the right of the gene identifier. A classification system is indicated as group labels to the right (E = eudicot, M = monocot). **B)** Unrooted phylogeny of the NRT2 family including twelve non-plant sequences identified as best GenBank BLAST hits using representative members from each plant group. Bootstrap values are given for central branches only up to the branch defining a group. Approximate locations of functionally characterized members discussed in the text are indicated.For database accession numbers of the 93 protein sequences included, see Additional file 1.

**Figure 3:**
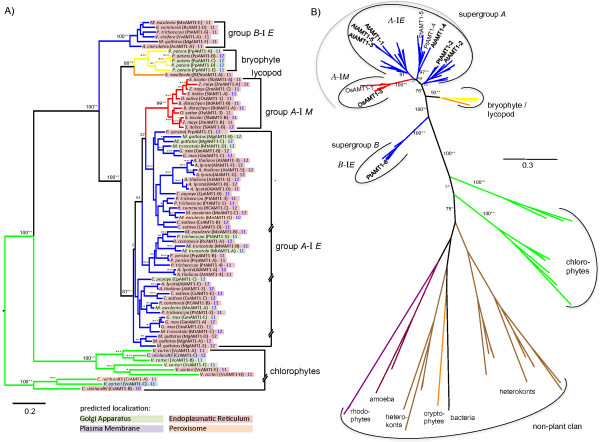
**Maximum Likelihood phylogenetic reconstruction of the AMT1 family.** Taxonomic groups are colored as in Figure 1. **A)** Rooted phylogram of the AMT1 family in plants only. Bootstrap values of the ML analysis are given and corresponding bootstrap values (>75%) from a distance neighbor joining and a parsimony analysis are indicated as green and red stars, respectively (within groups, bootstrap values >75% are displayed as stars only for all three analyses). Subcellular localization predictions, transmembrane topology predictions, and a classification system is indicated as described in Figure 2. **B)** Unrooted phylogeny of the AMT1 family including 21 non-plant sequences identified as best GenBank BLAST hits using representative members from each plant group. Bootstrap values are given for central branches only up to the branch defining a group.

**Figure 4:**
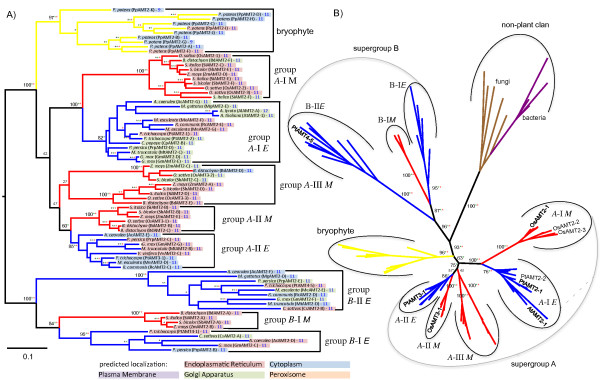
**Maximum Likelihood phylogenetic reconstruction of the AMT2 family.** Taxonomic groups are colored as described in Figure 1. **A)** Phylogram of the AMT2 family including plant sequences only supplemented with ML bootstrap values (NJ and parsimony bootstrap values (>75%) indicated as blue and red stars), subcellular localization predictions, topology predictions, and proposed classification system. The root of this tree was defined through the analysis shown in **B**). **B)** Unrooted phylogeny of the AMT2 family including eleven most similar non-plant sequences present in GenBank. Color-coding as described above. For database accession numbers of the protein sequences included, see Additional file 1.

Functionally characterized NRTs and AMTs [6,21,23-30] were used for BLASTP searches against the annotated proteomes derived from 20 land plant genome sequences and this set was complemented with two green algal species (both belonging to the *Chlorophyceae*) resulting in a total of more than 1,300 plant protein sequences analyzed (Table 1, Additional file 1). Sequences not named beforehand were given letters (e.g. PtNRT2-A, MgAMT1-B, etc.) and sequences that had been named or functionally characterized to some degree retained the original name assigned. The AMT1, AMT2, and NRT2 transporter classes are encoded by comparably small gene families in most plants ranging from one to 14 members. In contrast, the NRT1/PTR family can have more than 90 members (Table 1). Two *Chlorophyceae* genomes, from *Chlamydomonas reinhardtii* and *Volvox carteri*, were included as a root to the land plants. They contain NRT2 and AMT1 family members, but not AMT2s. A single NRT1/PTR like sequence is present in *V. carteri*, but not in *C. reinhardtii* (Table 1). When present, green algal and land plant sequences each form sister clades in rooted plant-only maximum likelihood phylogenetic reconstructions (part A of Figures 1, 2, 3, and 4) suggesting that a single NRT2 and AMT1 gene was present in the ancestor of *Viridiplantae*. To evaluate if all land plant sequences were indeed inherited vertically, we used representative members from all major clades in sequence similarity searches against GenBank excluding land plant and green algal species. If sequences with close homology to specific land plant sub-clades would exist outside the plant lineage (e.g. because they were transmitted horizontally) and be present in GenBank, similarity searches (such as BLAST) should identify them more readily than more distantly related vertically inherited sequences and they should be among the most similar hits. Inclusion of these non-plant sequences into the phylogeny should then place horizontally transmitted sequences within the plant clan, while vertically related sequences should form a distinct clan outside the whole plant family in unrooted phylogenies. In all four cases, all non-plant sequences formed a single clan (part B of Figures 1, 2, 3, and 4) suggesting that indeed all plant sequences are monophyletic and were inherited vertically.

**Table 1:** Members from the AMT1, AMT2, NRT1/PTR, and NRT2 gene families analyzed

		**Number of members analyzed**^**a**^			
**Taxonomic group**	**Species (abbreviation)**	**AMT1**	**AMT2**	**NRT1/PTR**	**NRT2**
Eudicot	*Aquilegia coerulea* (Ac)	1	4	48	2
	*Arabidopsis lyrata* (Al)	6	1	49	6
	*Arabidopsis thaliana* (At)	5	1	51	6
	*Carica papaya* (Cp)	2	1	41	2
	*Cucumis sativus* (Cs)	4	2	49	1
	*Glycine max* (Gm)	5	5	96	3
	*Manihot esculenta* (Me)	5	4	61	3
	*Medicago truncatula* (Mt)	4	3	52	1
	*Mimulus guttatus* (Mg)	6	2	52	7
	*Populus trichocarpa* (Pt)	6	5	70	6
	*Prunus persica* (Prp)	3	4	49	2
	*Ricinus communis* (Rc)	4	3	41	4
	*Vitis vinifera* (Vv)	1	1	44	0
Monocot	*Brachypodium distachyon* (Bd)	2	6	67	5
	*Oryza sativa* (Os)	2	6	65	3
	*Setaria italic* (Si)	2	6	74	7
	*Sorghum bicolor* (Sb)	2	6	67	4
	*Zea mays* (Zm)	3	5	51	3
Lycophyte	*Selaginella moellendorffii* (Sm)	1	0	31	2
Bryophyte	*Physcomitrella patens* (Pp)	5	10	18	8
Green algae	*Chlamydomonas reinhardtii* (Cr)	3	0	0	3
	*Volvox carteri* (Vc)	6	0	1	3

Given are the numbers of protein sequences included^a^ from each of the 22 fully sequenced *Viridiplantae* genomes studied. A full list of sequences and respective accession numbers are given in Additional file 1.

^a^Actual family sizes may be larger, because partial sequences and those leading to distortions in the phylogenies were excluded here.

Within the land plants, several gene birth and death events apparently occurred throughout the lineage, giving rise to a complex mixture of subfamilies. Clades were initially characterized as chlorophyte, bryophyte, lycophyte, or angiosperm. Within the angiosperms, ‘groups’ were defined where there was a single common ancestor between a eudicot and a monocot clade. Groups were combined to ‘supergroups’, if they separated prior to the embryophyte/bryophyte split, i.e. were separated by *P. patens* sequences.

### The NRT1/PTR family

The NRT1/PTR family is named after the functions of its founding members, namely nitrate transporters (NRT1) and peptide transporters (PTR). The gene family is comprised of 54 family members on average in land plants, ranging from 18 copies in the moss *Physcomitrella patens* to 96 copies in *Glycine max* (Table 1). Phylogenetic reconstructions covering a total of 1,077 plant and 24 non-plant sequences separated the family into ten supergroups (Figure 1), which were further separated into a total of 32 groups (Additional file 2). Beyond NO_3_^-^ and peptide transport, the functions in this family cover a wide range including glucosinolate transport [14], abscisic acid transport [13], and NO_3_^-^ excretion (AtNAXT1) [31]. To test whether these diverse plant sequences likely form a monophyletic group in plants, representative sequences from multiple groups within each supergroup were used in BLASTP searches against the GenBank database, excluding *Viridiplantae* sequences and the ten best hits each (lowest expect value) were retained. This resulted in a total of 24 proteins from diverse eukaryotes including animals, amoeba, and heterokonts (Additional file 1) sharing up to 32% overall sequence identity with plant sequences. Many of the animal sequences were annotated as SoLute Carrier 15 (SLC15) family proteins, and reciprocal BLAST searches using the human SLC15A1 protein, which encodes a peptide transporter [32], against the Phytozome database revealed NRT1/PTR family members as best hits. We therefore opted to name uncharacterized proteins as SLC15 followed by a gene identification letter (Additional file 1)^a^.

Inclusion of the non-plant sequences into the phylogeny placed none of the non-plant sequences within a plant clan and all non-plant sequences form a single clan in the unrooted phylogeny (Figure 1B). This suggests that, despite the variety of functions, the NRT1/PTR family appears to be monophyletic in plants.

Targeted analyses of the green algal (*C. reinhardtii* and *V. carteri*) genomes revealed a single protein from *V. carteri*. The *V. carteri* protein is located between the non-plant clan and the remainder of the *Viridiplantae* (Figure 1). This may suggest that the *V. carteri* genes shares a common ancestry with other *Viridiplantae* NRT1/PTRs. Although the second green alga analyzed here (*C. reinhardtii*) also belonging to the *Chlorophyceae* does not contain a homolog in its annotated genome, other green algae, including *Chlorella variabilis*, *Coccomyxa subellipsoidea* (both belonging to the *Trebouxiophyceae)*, and *Ostreococcus tauri* (*Mamiellophyceae*) do contain NRT1/PTR like sequences with high sequence similarity to the *V. carteri* gene included here (based on sequence similarity searches against their annotated proteomes available at the JGI).

Within the land plants, it is apparent that both NRT1s and PTRs are polyphyletic, i.e. functionally characterized NRT1s and PTRs are more closely related to functionally distinct proteins than to other proteins with identical function (Figure 1). Predicting the ancestral function of the family in land plants is difficult with the relative scarcity of functional data available, but given that the homologous SLC15 family in animals transports peptides and amino acids [32], it may be assumed that this is the ancestral function of the NRT1/PTR family. If this is true, a minimum of three independent NRT gain of functions must be assumed. On the other hand, assuming an ancestral NRT function would require a minimum of four PTR gains (or three gains plus one reversion back to NRT). But again, this speculative parsimony argument is based on a very small number of functionally characterized proteins in a large tree.

Despite the polyphyletic characteristics of the family and the relative dearth of functionally characterized members, it appears possible to define groups, or even supergroups that share common functions.

### Supergroup A

**Figure 5:**
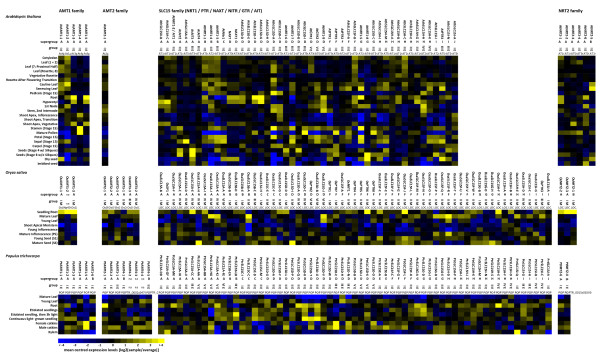
***In silico *expression profile of putative AMTs and NRTs.** Microarray data were obtained from The Bio-Array Resource for Plant Biology [81] for all family members from *A. thaliana*, *O. sativa*, and *P. trichocarpa*. Note, that AMT3s and AMT4s are members of the AMT2 family.

In supergroup *A*, AtNRT1-2 is quite distantly related to other *Arabidopsis* NRT1s present in the adjacent clan (supergroup *B*). *AtNRT1-2* has above average expression levels in floral organs and rosette leaves (Figure 5). In contrast, Huang *et al.*[33] reported high levels of *AtNRT1-2* expression in roots, primarily in root hairs and root epidermis. A transient repression of *AtNRT1-2* in response to NO_3_^-^ supply has been observed while *AtNRT1-1* (in supergroup *B*) expression increases [33]. AtNRT1-2 has NO_3_^-^ transport activity but no peptide transport activity [33]. It was a surprising observation that AtNRT1-2 also transports the plant hormone abscisic acid (ABA), indeed with greater affinity than NO_3_^-^ and was thus named AIT1 (ABA-Importing Transporter 1) [13]. Kanno *et al*. [13] suggest ABA import activity for three additional members of supergroup *A.* While AtAIT1/NRT1-2 and AtAIT2 are located in group *A*-II, AtAIT3 and AtAIT4 are contained in the sister-group *A*-III (Additional file 2). In total, six groups can be distinguished within supergroup *A*, but the only other functionally characterized member of supergroup *A* is OsSP1 from group *A*-I (Figure 1). OsSP1 is located in the plasma membrane and is needed for panicle elongation in rice [34,35]. Both voltage-clamp and yeast/bacteria mutant complementation failed to show nitrate transport activity indicating that it transports an alternate substrate [34]. Given the ABA transporting function of other supergroup *A* members, this alternative substrate could be ABA, consistent with the *sp1* phenotype showing a reduction in panicle size [34]. If this is the case, supergroup *A* may be an ABA transporter clan that may have evolved from an ancestral nitrate transporter. This speculation is congruent with the notion that supergroup *B*, forming an adjacent clan to supergroup *A*, contains four characterized NRTs (AtNRT1-1, AtNRT1-3, AtNRT1-4, and MtNRT1-3).

### Supergroup B

Distinct *A. thaliana* NRT1s define one of the three different groups present in supergroup *B* and MtNRT1-3 shares group *B*-III with AtNRT1-3 (Figure 1, Additional file 2). *AtNRT1-1* is largely constitutively expressed and encodes a dual-affinity NO_3_^-^ transporter, performing both low-affinity and high-affinity transport, mediated by phosphorylation [36]. AtNRT1-1 has been described as a NO_3_^-^ sensor [37]. It functions in stomatal opening [38] and in regulation of AtNRT2-1 [39]. MtNRT1-3 is also a dual affinity transporter and is involved in primary root growth, NO_3_^-^ sensing, and is developmentally regulated in an N-dependent manner in roots [40]. *AtNRT1-3* has high expression levels in many tissues with highest expression in late stage flower petals and sepals (Figure 5). *AtNRT1-3* has inducible expression only in shoots but is repressed in roots [30]. AtNRT1-4 (defining group *B*-II) has NO_3_^-^ transport activity in *Xenopus* oocytes [41]. It has non-inducible expression in the roots, but may be NO_3_^-^ induced in shoots [30,41]. *AtNRT1-4* has highest expression levels in leaf tissues based on microarray analyses (Figure 5) consistent with its known expression in petioles and leave midveins [41]. This, together with the phenotype of the *Atnrt1-4* mutant, implies an important role in NO_3_^-^ redistribution and homeostasis within the plant [41]. Together, there is strong evidence that all groups within supergroup *B* contain *bona fide* nitrate transporters, suggesting that supergroup *B* is NRT exclusive. However, most members have not been functionally characterized leaving the possibility of additional functions.

### Supergroup C

Supergroup *C* contains two functionally characterized members, both nitrite (NO_2_^-^) transporters (NiTR; Figure 1). AtNiTR1 from *A. thaliana* and CsNiTR1 from *C. sativus* each define the two groups present within supergroup *C* (Additional file 2). CsNiTR1 (group *C*-II) mediates NO_2_^-^ efflux when expressed in yeast, is localized to chloroplast membranes and may load cytosolic NO_2_^-^ into the chloroplast stroma [42]. AtNiTR1 is a member of group *C*-I, and *Atnitr1* knockout mutants accumulate NO_2_^-^ in leaves, suggesting a similar role to CsNiTR1 [42]. Together this suggests that the primary function of supergroup *C* is nitrite rather than nitrate or peptide transport. Supergroup C shares a common origin with supergroup D (Figure 1) and each supergroup is supported with high bootstrap values. However, the relative position of the single *P. patens* and the four *S. moellendorffii* sequences separating the supergroups are not well-resolved, precluding placement to the base of either supergroup *C* or *D*. The addition of more non-vascular and basal vascular species may resolve this part of the phylogeny.

### Supergroup D

Supergroup *D* highlights the polyphyletic relationship of the N-transporting proteins present in the NRT1/PTR family. It contains groups characterized as glucosinolate transporters (GTR), NO_3_^-^ excretion carriers (NAXT) and NO_3_^-^ transporters (NRT1), the latter being separated from NRT1s in supergroup *B* by the nitrite transporters present in supergroup *C* (Figure 1). The NO_3_^-^ excretion (NAXT) group *D*-I forms a basal clan in supergroup *D*. This group contains one characterized NAXT protein, but Segonzac *et al*. [31] identified six additional sequences as putative NAXTs all present in group *D*-I. NAXT1 is plasma membrane localized in the cortex of mature roots [31]. NO_3_^-^ efflux capabilities of the NAXT proteins were demonstrated *in vitro*, and RNAi transgenic plants accumulate NO_3_^-^ in roots [31]. As there are no other characterized types of transporters present in this clan, group *D*-I may define a NAXT-exclusive group.

Group *D*-IV (Additional file 2) is one of only two groups in the family containing two different types of transporters: the nitrate transporter AtNRT1-9 and the glucosinolate transporters AtGTR1 and AtGTR2 [14,43]. AtGTR1 is localized to the vascular tissue in leaves and can transport 4-methylthiobutyl glucosinolate. AtGTR1 likely performs a role in distributing glucosinolates within the leaf, possibly performing an import function into glucosinolate-rich cells adjacent to the phloem [14]. AtGTR2 transports 4-methylthiobutyl glucosinolate at 75% the rate of AtGTR1, is localized to veins in leaves, and likely performs a major role in apoplastic phloem-loading of glucosinolates [14]. The two AtGTRs and AtNRT1-9 form a closely related group of *Arabidopsis* paralogs within group *D*-IV (Additional file 2). AtNRT1-9 has highest expression in roots and stems (Figure 5) and has NO_3_^-^ transport activity in *Xenopus* oocytes [43]. It is not rapidly induced upon NO_3_^-^ supply; however, expression levels are increased over long-term exposure to NO_3_^-^. AtNRT1-9 is plasma membrane localized and expressed in the companion cells of phloem in roots [43]. Combined with the observation that *Atnrt1-9* knockout mutants have reduced NO_3_^-^ concentrations in phloem, this provides strong evidence that AtNRT1-9 is responsible for phloem loading of NO_3_^-^. Interestingly, AtNRT1-9 also has minor glucosinolate transport activity [14]. These data suggest that *GTRs* have evolved recently within the *Brassicaceae* lineage from *AtNRT1-9*[14]. The relatively long branch length towards AtGTR1/GTR2 may support this, although tests for signatures of positive selection would be needed.

Within group *D*-III, *AtNRT1-6* has highest expression levels in seeds (Figure 5). AtNRT1-6 is not responsible for root uptake of NO_3_^-^ as it is only expressed in reproductive tissues [24]. AtNRT1-6 confers low-affinity NO_3_^-^ transport and it has been suggested that AtNRT1-6 plays a role in transporting NO_3_^-^ from maternal tissue to the developing embryo [24]. AtNRT1-7 is also a confirmed low affinity NO_3_^-^ transporter expressed primarily in leaves, in particular minor veins [44], and floral organs (Figure 5) suggesting a role in phloem loading of NO_3_^-^.

### Supergroup E and supergroup J

Both supergroups E and J are comparatively small and contain no functionally characterized members. Supergroup E contains a single copy from most species analyzed and is rooted by a single *P. patens* and two *S. moellendorffii* sequences (Additional file 2) suggesting that it has an essential function in plants. In contrast, supergroup J contains exclusively eudicot sequences and only seven out of 13 eudicot species maintain members in this supergroup. It appears supergroup J members have evolved specialized functions in species in which they are maintained.

### Supergroup F and G

A well-supported clan contains a clan comprising all non-plant sequences, the green algal sequence from *V. carteri*, and several angiosperm clans. Among them, supergroup G is supported with strong bootstrap support and is rooted by multiple lycopod and bryophyte sequences (Figure 1). The remaining angiosperm sequences form multiple clans, many of which lack bootstrap support and are not maintained in the sub-phylogeny containing only plant sequences (Additional file 2). This prevents defining separation times and we therefore refrained from defining additional supergroups and instead combined these sequences into a single, paraphyletic supergroup. This supergroup *F* contains mainly PTRs but also a characterized NRT and, together with supergroup *G* NRT1s, is the clan most closely related to the non-plant sequences included (Figure 1). This supergroup contains three groups, two of which (*F*-I and *F*-III) are well supported. The third (*F*-II) contains one eudicot clan and four monocot clans (Additional file 2). This clan also contains the sole green algal and a single lycopod sequence, and a monocot clan adjacent to the non-plant sequences in the unrooted phylogeny (Figure 1). However, very poor bootstrap support separating the monocot clans precludes defining if they separated before or after the monocot/eudicot split and thus they were all grouped together into the paraphyletic group *F*-II*M* here. At least eight gene duplications must be assumed prior to separation of the monocot species analyzed (Additional file 2). The two characterized members in group *F*-II*M* are OsPTR6 and OsNRT1 from *O. sativa. OsPTR6* confers peptide transport as it is capable of transporting Gly-His and Gly-His-Gly di- and tri-peptides [45]. Also the sole characterized member in the eudicot *F*-II*E* group is a peptide transporter. AtPTR2 was the first di-/tripeptide transporter identified in *Arabidopsis*[46-49] with high mRNA expression levels in germinating seed, root, and young leaf tissues [47]. *AtPTR2* antisense transgenic plants displayed delayed flowering time and arrested seed development [48,50]. In contrast, the third characterized member of the *F*-II group encodes a NO_3_^-^ transporter. OsNRT1 is constitutively expressed in the root epidermis and in root hairs [51]. Like most other NRT1s, OsNRT1 is a low-affinity transporter [35,51]. Despite the fact that OsNRT1 is more closely related to PTRs than to other NRT1s (Figure 1), Lin *et al*. [51] observed no peptide transport. This is another example of transporters with distinct functions sharing the same group. In contrast, group *F*-I (Additional file 2) appears to be a PTR-exclusive group. AtPTR1 transports di-/tripeptides with low selectivity, is expressed in vascular tissue throughout the plant, and likely performs a role in long distance transport [49]. AtPTR5 mediates high-affinity transport of dipeptides and likely supplies peptides to maturing pollen, developing ovules, and seeds [52]. Interestingly, overexpression of *AtPTR5* resulted in enhanced shoot growth and increased N content [52].

Supergroup *G* is nested within the larger, unresolved *G*/*F*/non-plant clan, but is separated by bryophyte sequences and is supported by high bootstrap support across all three methods employed. The clan has therefore been designated a separate supergroup. Supergroup *F* appears to be NRT-exclusive (Figure 1). The defining members, *AtNRT1-5* and *AtNRT1-8* have above average expression levels in seeds, but *AtNRT1-5* also has relatively high expression levels in roots, flowers and senescing leaves (Figure 5). *AtNRT1-5*, like *AtNRT1-1* (in supergroup *B*), is NO_3_^-^ inducible and strongly expressed in roots; however the response of *AtNRT1-5* to NO_3_^-^ supply is much slower than that of *AtNRT1-1*[53]. Both AtNRT1-5 and AtNRT1-8 have confirmed NO_3_^-^ transport activity [53,54]. AtNRT1-8 has high expression levels in xylem parenchyma cells in the stele and *Atnrt1-8* mutants have increased concentrations of NO_3_^-^ in the xylem, suggesting that AtNRT1-8 is responsible for removing NO_3_^-^ from the xylem [54]. Thus, AtNRT1-5 and AtNRT1-8 appear to define a group (*D*-I *E*) responsible for movement of NO_3_^-^ within the plant. None of the other groups contain characterized members precluding judgment of functional diversity within superfamily *G*.

### Supergroup H and supergroup I

Supergroups H and I are adjacent clans containing only one functionally characterized member, AtPTR3 (in supergroup I). *AtPTR3* is induced in vegetative tissues by histidine, leucine and phenylalanine and upon salt stress. Germination frequency of *ptr3* mutants was reduced on salt-containing media [55]. *AtPTR3* is also induced upon mechanical wounding, and *ptr3* mutants have increased susceptibility to virulent pathogenic bacteria suggesting that AtPTR3 has a general role in stress response [55]. Supergroup I may be PTR specific; however, basing this hypothesis on only one functionally characterized protein is premature. Notably, supergroup *H* is one of the largest supergroups within the NRT1/PTR family but lacks any characterized members, highlighting the need for further functional characterizations.

In summary, it appears that within the NRT1/PTR family, evolutionary distinct clans define functionally distinct groups in many, but notably not all cases. Our classification is largely in agreement with Plett *et al.*[56], who analyzed the NRT1 family in select monocots and eudicots: Supergroup A (containing AtNRT1-2), supergroup B (containing AtNRT1-1, AtNRT1-3, and AtNRT1-4), supergroup D (containing AtNRT1-6 and AtNRT1-7), and supergroup G (containing AtNRT1-5 and AtNRT1-8) were also indicated [56]. The additional supergroups defined here arise from the large number of species analyzed, and because Plett *et al*. [56] intentionally did not include characterized PTRs.

### The NRT2 family

The NRT2 gene family is comprised of one to eight family members with 11 or 12 predicted TM domains. The majority of NRT2 family members are predicted to be localized to the peroxisome or cytoplasm (Figure 2). It is important to note that localization prediction of hydrophobic membrane-spanning proteins is particularly challenging. Especially cytoplasmic localization may be interpreted with caution as this is based on the apparent absence of a localization signal. Based on the trans-membrane structure prediction, all these proteins are obviously targeted to a membrane. Phylogenetic reconstructions suggest two distinct clans of NRT2s in angiosperms each containing a single group proper (Figure 2). Also Plett *et al.*[56] noted separation of the NRT2 family prior to the separation of monocots and eudicots. Inclusion of bryophyte and lycopod sequences suggests that these groups separated early in vascular plant evolution, but after the separation of bryophytes and tracheophytes. Therefore, we defined them as different groups, but not as different supergroups, which would require separation prior to the bryophyte/tracheophyte split. However, bootstrap support for this placement is low and conflicting results were obtained with alternative methods (distance and parsimony). Thus, the separation time of group I and group II remains unresolved, but clearly happened early in embryophyte or tracheophyte evolution.

Inclusion of non-plant homologs (from the red alga *Pyropia yezoensis*, the heterokont brown alga *Ectocarpus siliculosus*, and ten bacterial sequences) resulted in a single non-plant clan separated by a long branch from the green algal and land plant sequences (Figure 2B). This is consistent with a monophyletic origin of *Viridiplantae* NRT2s shown earlier [57,58]. We thus rooted the plant only phylogeny (Figure 2A) with the green algal clan.

The bryophyte clade contains all *P. patens* NRT2s and is basal to the vascular plant clade. In the bryophyte clade, multiple gene duplications led to a total of eight *P. patens* NRT2s. In the lycophyte clade, there has been one duplication event leading to two copies of NRT2 in *S. moellendorffii*. None of these genes have been functionally characterized.

As there are only a few angiosperm species represented in group II (eight out of the 20 land plant species analyzed), it appears that gene loss in this group is common. There are both monocots and eudicots present in group II, suggesting that, contrary to AMT1 and AMT2 (described below), the function performed by group II NRT2s is shared across eudicots and monocots. Within this group, *AtNRT2-5* has maximal expression in senescing leaves based on microarray data (Figure 5), but is described as being nitrate repressible, expressed in roots and shoots, and, contrary to root-uptake NRT2s, having maximum expression in the absence of NO_3_^-^[30]. This may indicate a function in remobilization of NO_3_^-^ from stored pools [59]. While not functionally characterized, the expression profile for *PtNRT2-X* (group II) shows maximal expression in male catkins. Together, this may suggest that eudicot group II NRT2s fulfill functions in NO_3_^-^ remobilization within the plant rather than having root uptake activities. In contrast, the rice *OsNRT2-1* (in group II *M*) has maximal expression in seedling roots, is NH_4_^+^ repressible, and, as found for typical root-uptake NRT2s, is up-regulated in response to low concentrations of NO_3_^-^[60]. This supergroup thus contains *bona fide* NRTs that may be responsible for both root uptake and within plant mobilization of NO_3_^-^; however more functional data is required to separate these functions in an evolutionary context.

The distinct group I contains members from all angiosperm species analyzed. Consistent with Slot et al. [58], who included only group I angiosperm sequences, monophyly of the group and the monocot and eudicot sister clades within the group are well supported (Figure 2). Group I *E* has undergone extensive gene amplification in all angiosperms analyzed except in *M. truncatula*, which contains only one NRT2. Within group I *E*, two sets of *Arabidopsis* paralogs can be identified: one comprising AtNRT2-1, -2, and -4, and the other containing AtNRT2-3 and -6, each alongside their *A. lyrata* orthologs (Figure 2). Obvious differences in expression patterns (Figure 5), both within and between these groups suggest different functions of individual members. AtNRT2-1 and AtNRT2-2 are responsible for HATS transport in roots [61,62]. *AtNRT2-1* is induced upon supply with low levels of NO_3_^-^ and also directly regulates lateral root formation under N-limiting conditions [63]. A knockout mutant of either *AtNRT2-1* or *AtNRT2-2* results in a reduction of iHATS; however, a double knockout is required to reduce the cHATS, suggesting partially redundant functions of these paralogs [62]. Furthermore, *AtNRT2-4* is over-expressed in *Atnrt2-1/Atnrt2-2* double knockouts, suggesting compensation; but over-expression of *AtNRT2-4* does not restore NO_3_^-^ transport in the double mutant [59]. Thus, it is likely that AtNRT2-4 is responsible for the re-mobilization of stored NO_3_^-^, but it cannot replace root uptake functions of AtNRT2-1 and -2.

AtNRT2-3 and AtNRT2-6 are less well characterized but *AtNRT2-3* expression is slightly responsive to NO_3_^-^ supply both in shoots and roots [30] and *AtNRT2-6* has very low overall expression with highest expression in roots. AtNRT2-6 may be responsible for very high affinity NO_3_^-^ transport [59]. In conclusion, individual group I *E* members in *A. thaliana* fulfill distinct physiological functions and even close paralogs (e.g. *AtNRT2-1* and -*2*) have only partially redundant functions. It may thus be expected that functional ‘radiation’ within this group has led to the maintenance of multiple copies with diverse functions in other species, also.

The monocot sister clade, group I*M*, has undergone multiple gene duplications at different times in its evolutionary history (Figure 2). The majority of group I *M* proteins are predicted to be localized to the cytoplasm, but five proteins are predicted to be localized to the secretory pathway, one to the golgi and four to the ER (Figure 2). Both rice NRT2s, *OsNRT2-2* and *OsNRT2-3*, have maximal expression in seedling roots (Figure 5) and *OsNRT2-2* is rapidly induced in roots upon NO_3_^-^ supply, then down-regulated quickly [25]. *OsNRT2-3* has a transient inducible response to NO_3_^-^, and, Yan *et al.*[64] have shown that OsNRT2-1, OsNRT2-2, and OsNRT2-3, like the NRT2s in *A. thaliana*, must interact with an additional protein, OsNAR2-1, to perform transport activity.

### The AMT1 family

The AMT1 gene family is comprised of 1–7 family members with either 11 or 12 predicted TM domains. Most AMT1 family members are predicted to be localized to the secretory pathway, namely endoplasmic reticulum (ER) or golgi apparatus (Figure 3). Phylogenetic reconstructions suggest the existence of two evolutionarily distinct clans of AMT1 members in angiosperms (supergroups *A* and *B*), which are separated by sequences from lycophytes and bryophytes (Figure 3). All *P. patens* sequences and the sole *S. moellendorffii* sequence form a monophyletic group at the base of supergroup *A*.

Two paraphyletic clans are apparent in the green algae and in each clan gene duplications prior to speciation generated the copies present in *V. carteri* and *C. reinhardtii*. No functional data are available for any of these green algal AMT1 transporters. McDonald et al. [16] and McDonald et al. [57] provide extensive evolutionary analyses of the AMT1 family across multiple green algal and other eukaryotic lineages. Both identified a single land plant clade rooted by green algal clades. Here, we expanded land plant coverage and identified a new divergent land plant clade clan (supergroup B). In order to validate that also supergroup B sequences are part of the same land-plant clade identified previously [16,57], we used representative members of each group to identify the ten most similar non-plant members present in GenBank. This resulted in an overlapping set of 18 sequences from bacteria, heterokonts, cryptophytes, amoeba, and rhodophytes (Additional file 1). Upon inclusion of the non-plant sequences into the phylogeny, all land plant AMT1s remained in a single clan separated by green algal sequences from the non-plant clan (Figure 3B) suggesting that all plant AMT1s analyzed here were inherited vertically from a common ancestor and are part of the single land plant clan identified by McDonald *et al.*[16]. Thus, supergroup *A* AMT1s most likely separated from supergroup *B* AMT1s prior to the bryophyte/embryophyte split, but after separation of land plants and green algae.

We rooted the plant only phylogeny (Figure 3A) with green algal sequences. No bryophyte, lycophyte, or monocot homologs to the eudicot *B*-I *E* members are present in extant species analyzed. Also, most eudicot species analyzed here do not contain supergroup B members, including all species within the *Brassicales*, suggesting a specialized function of these ancient AMT1s in those species that maintained them. Biochemical functional characterization of any supergroup *B* AMT1s is lacking, but *PtAMT1-6* expression is increased upon ectomycorrhizal symbiosis [6]. This may indicate that members of this supergroup *B* perform symbiont-related transport, such as NH_4_^+^ uptake from mycorrhizae.

Within supergroup A, a single, well supported monocot clade and multiple eudicot clades are apparent. Bootstrap support for deciphering the relationship between these eudicot clades and the single monocot clade is missing. Therefore it cannot be determined whether the clades separated prior of after the separation of monocot and eudicots. For this reason, all eudicot sequences within supergroup *A* were combined in a single group, named A-I*E*. Extending taxonomic depth may be necessary to determine if some of these clades actually define separate groups. Most *A*-I AMT1s are predicted to be ER localized, but eight proteins may be golgi apparatus localized and one has a peroxisome prediction. Both clades can each be further divided into subclades that contain members from all species in the respective group. Subsequent duplications are apparent in both lineages, but the group is particularly expanded in eudicots (Figure 3) where at least three subclades exist. Additional recent duplications gave rise to groups of paralogs in multiple species including *A. thaliana*. Thus, the vast majority of AMT1s amplified separately in eudicots and monocots suggesting relatively recent functional diversification.

The two rice AMT1s included belong to group *A*-I *M*. Also the third rice AMT (OsAMT1-2) groups within this clade, but was excluded from the final analyses due to a very long branch towards OsAMT1-2 that has the potential to distort the overall topology of the phylogeny. Despite this, all three encode ammonium transporters validated by complementing an ammonium uptake-deficient yeast strain [65]. All show maximal transcript levels in seedling roots (Figure 5). Both *OsAMT1-1* and *OsAMT1-2* are ammonium induced in N-starved plants [65], and are repressed by transfer from low to high ammonium, which correlates with high affinity NH_4_^+^ uptake [29]. Kumar *et al*. [29] showed that *OsAMT1-3* (in a separate *A*-I *M* subclade) transcript levels remained largely unaffected by such treatments suggesting functional divergence within the monocot *A*-I *M* subclades.

The paralog group in *Arabidopsis* contains the well-characterized *A. thaliana* AtAMT1-1, AtAMT1-3 and AtAMT1-5, and five *A. lyrata* orthologs. *AtAMT1-1* is expressed in the rhizoderm and root cortex [28] while *AtAMT1-2* is expressed in root endodermal cells [66]. AtAMT1-1 and AtAMT1-3 are plasma membrane localized, and all three are high affinity NH_4_^+^ transporter proteins and involved in root-uptake of NH_4_^+^ in an additive manner [28,67]. While *AtAMT1-3* and *AtAMT1-5* are root specific, *AtAMT1-1* is expressed more broadly, including roots, leaves, and sepals (Figure 5). In addition to its function in root NH_4_^+^ uptake, AtAMT1-3 has a regulatory function in NH_4_^+^-induced lateral root branching [68]. In contrast, the more diverse *AtAMT1-4* (in a distinct clade in group *A*-I *E*) is not expressed in roots, but is specifically expressed in pollen. It also encodes a plasma membrane localized high-affinity NH_4_^+^ transporter [23]. The *P. trichocarpa* AMT1s sharing a clade with AtAMT1-4 (*PtAMT1-4* and *PtAMT1-5*) are also expressed in male and female flowers, and, in the case of *PtAMT1-4*, in leaves [69] (Figure 2). Taken together, this suggests distinct physiological functions for *A*-I *E* subclades in root uptake or reproductive organ supply of ammonia.

The fifth *A. thaliana* AMT1 family member, *AtAMT1-2,* belongs to the third *A*-I *E* clade (Figure 3) and encodes a transporter that mainly contributes to the HATS. It is expressed in young root endodermal cells and more mature cortical cells, but is not induced by low nitrogen availability [19,67]. In addition to roots, *AtAMT1-2* is also expressed in flowers and stem nodes with maximal expression in cauline leaves (Figure 5). Likewise, its *P. trichocarpa* ortholog, *PtAMT1-2*, has high levels of expression in roots [69], but also in other tissues such as seedlings grown in continuous light and male catkins (Figure 5). *PtAMT1-2* is induced by ectomycorrhizal symbionts together with *PtAMT1-4* and *PtAMT1-6* (named *PtAMT1-3* in Selle *et al.*[69], but *PtAMT1-6* in Couturier *et al*. [6] and at ‘Phytozome’). The *P. tremula x tremuloides* ortholog of PtAMT1-2 encodes a high affinity transporter with similar expression patterns [69]. Together, this may suggest broader functions for members of this clade in within-plant and plant-symbiont ammonium distribution rather than high affinity uptake from the soil.

In summary, it is obvious that in both eudicots and monocots, early gene duplication events generated the supergroup *A* AMT1 subclades. Expression profile and physiological differences of subclade members indicate functional diversifications in individual species, but more detailed information from more species is necessary to generalize functional diversifications to the subgroups identified. This is especially true for the more divergent supergroup *B* members.

### The AMT2 family

The AMT2 gene family is comprised of 1–10 family members with the vast majority predicted to possess 11 TM domains (Figure 4). Unrooted phylogenetic reconstructions suggest there are two major clans of AMT2s in angiosperms forming supergroups. All *P. patens* sequences form a single bryophyte clan (Figure 4B). It appears that many duplication events occurred in the bryophyte lineage, both ancient and more recent, leading to ten copies of AMT2 in *P. patens*. Green algal genomes analyzed here (both belonging to the *Chlorophyceae*) do not contain genes with sequence similarity to AMT2s (Table 1). Extended sequence similarity searches targeting the *Chlorophyta* returned exclusively sequences from *Mamiellales*. McDonald *et al.*[57] focused on these AMT2s present in *Mamiellales* and showed that they do not share an immediate evolutionary history with land plant AMT2s. Instead, land plant AMT2s likely arose from a horizontal gene transfer (HGT) event [16,57]. Our BLAST searches using representative members from all groups against the GenBank database excluding *Viridiplantae* revealed several bacterial species including the extremophile chemoautotrophic bacterium *Leptospirillum rubarum* and the chemolithotroph *Acidithiobacillus caldus* as most similar sequences (Additional file 1). McDonald *et al.*[57] and McDonald *et al.*[16] identified the same bacterial species as intermediate between typical *Archaea* AMT2s and land plant AMT2s (referred to as MEPα in [16]). Most other bacterial genomes lack this type of AMT2, thus it has been argued that the land plant AMT2 likely arose through a HGT event from a member of *Archaea* possibly via a gamma proteobacterial intermediate host [16,57]. The same host is also the likely origin of AMT2 (MEPα) sequences from fungi found only in the leotiomyceta [16]. Consistently, proteobacterial and fungal sequences were among the best BLAST hits, and this group of non-plant sequences formed a separate clan when included into the phylogeny (Figure 4B). This confirms that also the extended set of plant sequences used here belong to the same monophyletic land plant clade identified earlier [16,57].

Within the plant clan, angiosperm sequences form two clans separated by a bryophyte clan, suggesting that the common ancestor of bryophytes and angiosperms possessed two AMT2 genes and that one copy was lost in the bryophytes. We thus placed the root of the plant phylogeny between supergroup B and the bryophytes (Figure 4A). The ancient separation of supergroups *A* and *B* AMT2s in angiosperms, together with the fact that both were maintained in all angiosperm species analyzed, clearly suggests a functional difference. However, in depth characterizations of AMT2s are scarce.

Supergroup *A* contains three groups, one of which (group *A*-III*M*) was maintained in monocots only. However, group *A*-III*M* contains two distinct clades and bootstrap support for them forming a monophyletic group together is poor (Figure 4A). Extending species depth may thus split this group into two separate monocot groups. None of the group A-III*M* members has been functionally characterized. OsAMT3-3 and OsAMT3-2 (in distinct group *A*-III *M* clades) have high expression levels in the seedling root; however, *OsAMT3-3* (*A*-III) is also expressed to high levels during early seed development (Figure 5).

Group *A*-I members, present in monocots and eudicots, are largely predicted to be localized to the endomembrane system. Recent duplications gave rise to paralogs present in *P. trichocarpa*, *M. esculenta*, and *G. max*. The remainder of the species contain only a single type *A*-I AMT2 gene (Figure 4). Of the eudicots in group *A*-I, *PtAMT2-1* has nearly exclusive expression in roots and encodes confirmed ammonium transport activity, shown through complementation of MEP (MEthylammonium transPorter) deficient yeast [6]. PtAMT2-2 has also been shown to have NH_4_^+^ transport activity as well as detectable expression in roots [6] in addition to high expression in male catkins (Figure 5). The sole AMT2 gene in *A. thaliana* (*AtAMT2-1*) belongs to group *A*-I *E* and has maximal expression in the stem internodes as well as notable expression levels in leaves and flowers, based on published microarrays (Figure 5). Sohlenkamp *et al.*[70] also noted expression in roots. AtAMT2-1 has ammonium transport activity similar to that of AtAMT1-1 at pH 7.5, but transport capacity of AtAMT2-1 is an order of magnitude lower than that of AtAMT1-1 at pH 6.5 [70].

The monocot group *A*-I appears to have undergone an early duplication event preceding speciation in the monocots, leading to two sister clades (Figure 4A). *OsAMT2-1* is expressed fairly broadly in both roots and shoots [71] (Figure 5). OsAMT2-1 has NH_4_^+^ transport activity in yeast complementation tests, at least at high N concentrations [71]. *OsAMT2-3* appears more specifically expressed during inflorescence development and late stages of seed development, while *OsAMT2-2* shows high expression in the seedling root (Figure 5). *OsAMT2-2* transcripts are induced upon supply of NH_4_^+^[27], and have maximal expression levels in seedling roots, suggesting a role for OsAMT2-2 in NH_4_^+^ uptake from the soil.

In eudicots, there is either only a single copy *A*-II AMT2 present, or type *A*-II sequences are absent from eudicot genomes, as is the case in *A. thaliana*. Functional characterization of group III AMT2s is limited to *PtAMT3-1*, which has maximal expression in male catkins and has virtually no expression in roots (Figure 5). *PtAMT3-1* is induced during senescence, but whether it functions as an NH_4_^+^ transporter remains unclear, as the gene is unable to complement MEP deficient yeast [6]. Of the monocot genes in group *A*-II, OsAMT3-1 has expression in many tissues, but highest expression in seeds. Generally, *OsAMT3-1* has much lower expression levels than OsAMT2-1 (in group *A*-I) both in in roots and shoots [71].

In summary, the only biochemically characterized AMT2s reside in group *A*-I and given the lack of MEP complementation and/or the lack of functional analyses for members from any other group, it remains to be shown that transporters of the other groups are indeed NH_4_ transporters, or instead transport other solutes.

## Conclusion

We here provide a comprehensive evolutionary view of ammonium, nitrate, and peptide transporter families across a large number of land plant species. This enables a phylogentic classification of each family and affords a foundation for further functional characterization. Given the depth of species coverage, it can be assumed that most, if not all, groups of N-transporters in angiosperms have been defined. All four families of N-transporters appear to be inherited vertically within the land plants, although evolutionary distinct, sometimes small and lineage specific groups are obvious, suggestive of lateral gene transfer. These lineage specific groups likely separated prior to the bryophyte/tracheophyte split and were maintained only in select species suggesting specialized functions. McDonald *et al.*[57] also suggested monophyly of the land plant clades for all four N-transporter families, but given the broader scope of their study, it did not aim to resolve the evolutionary history within land plants. However, at least two HGT events within green algal AMT2s were evident in that study, one of which led to the monophyletic AMT2s in land plants.

Early divergence and extensive amplification is particularly obvious in the NRT1/PTR family. Ten supergroups were defined that separated prior to the bryophyte/tracheophyte split and subsequently underwent duplications, giving rise to at least 32 groups that separated prior to the monocot/eudicot split. This is paralleled with functional divergence in this family with four known substrates being transported, namely nitrate, peptides, abscisic acid, and glucosinolates. The most similar non-plant sequences encompass the solute carrier family 15 (SLC15) of animals. Given that SLC15 proteins are peptide transporters [32], it appears plausible that the ancestral function of the plant family was transport of organic N-containing solutes. Thus, it is obvious that nitrate transport activity is polyphyletic and evolved several times independently within the NTR1/PTR family. There appear to be multiple cases in which functional labels can be applied to groups proper within each supergroup. However, these functional assignments can only be tentative, given the paucity of functionally characterized proteins relative to the abundance of sequences analyzed, and given that NRT1s within at least two supergroups have clearly evolved to transport distinct substrates: N-containing glucosinolates and N-free isoprenoids, ie abscisic acid [13,14].

Subcellular localization predictions largely support the notion of functional divergence among discrete groups and subgroups. Functional information can be inferred from localizations, for instance, the extensive endomembrane system (golgi, ER, and plasma membrane) prediction in the AMT2 family may indicate primary localization to the plasma membrane, suggesting cellular uptake rater than intracellular compartmentalization of ammonium. However, some of the localization is unexpected, such as the high degree of peroxisome localization in the NRT2 family. This could be due to difficulties in predicting hydrophobic, membrane bound proteins, but also to the immaturity of proteome annotations, many of which are based on genomes recently released; but distinct functions in unexpected organelles should not be precluded.

Currently, no systematic nomenclature of the NO_3_^-^ and NH_4_^+^ transporters exists. Here, we suggest a naming system that pertains to group membership, defined as being derived from a single gene present in the last common ancestor of monocots and eudicots. This simple rule allows for easy addition of future sequences to groups, and formation of new groups, should the need arise.

Given the depth of angiosperm sequences available, we were able to dissect this taxonomic group comprehensively. However, it is apparent from the inclusion of *P. patens* and *S. moellendorffii* that a similar diversity also exists in non-seed plants, and that inclusion of additional taxa in these groups and other taxonomic groups, from ferns to gymnosperms, is necessary to assess the full evolutionary history of the N-transporting systems in all plants.

## Methods

### Sequence acquisition

Individual sequences and accession numbers from functionally characterized NRTs and AMTs were obtained through primary literature research [6,21,23-30]. These protein sequences were used in BLASTP searches against the *A. thaliana*, *P. trichocarpa*, *O. sativa*, and *Zea mays* proteome annotations using Phytozome [72] (http://www.phytozome.net/) and Genbank (http://www.ncbi.nlm.nih.gov/genbank). Sequences obtained from the initial BLAST searches were then used as query sequences against all organisms present on Phytozome as of January 10th, 2011. Over 1,300 sequences in total were obtained and are summarized in Additional file 1. Sequences that are putative transporters are given letters (PtNRT1-A, PtNRT1-B, etc.) and sequences that are functionally characterized to some degree retain the name they were given in the paper in which they were identified. The protein BLAST algorithm parameters used were BLOSUM62 comparison matrix, default word length of 3, allow gaps (existence cost of 11 and extension cost of 1), and included a filter of low complexity regions. Sequences were accepted from BLAST results as long as they were not a series of small fragments, shared at least 30% identity, and had an expect threshold lower than 1e^-50^.

### Alignments and phylogeny construction

The sequences for each family were aligned using DiAlign [73] using the Mobyle Portal [74]. The DiAlign program provides a scoring system based on local similarity of aligned blocks that indicates the alignment quality at each position. We excluded all positions that had a diagonal similarity of <40%.

Approximate maximum-likelihood phylogenetic reconstructions were generated using FastTree version 2.1 [75]. These phytogenetic reconstructions were generated based on the Jones-Taylor-Thornton model; models available in FastTree were evaluated using ProtTest [76].

Phylip’s SEQBOOT was used to generate resampled alignments, and phytogenetic reconstructions were generated for 1,000 replicates. Bootstrap values were then mapped to each node in the original phytogenetic reconstruction as fraction of times that split is maintained in the resampled tree. Supporting phylogenetic reconstructions using distance and parsimony methods were generated using the Phylip package [77]. Neighbor-joining trees were generated based on distance matrices using the Jones-Taylor-Thornton model. The resampling method was bootstrapping and consisted of 1,000 replicates. Phylogenies were visualized and rooted in FigTree [78] using green algae or *P. patens* sequences.

While analyzing initial phylograms, any sequences with especially long branches were investigated in the original alignment. If the sequence had large gaps exceeding more than 30% of the alignment length, or contained areas of extensive differences throughout the sequence (likely indicating a gene modeling artifact), it was excluded and the remaining sequences were re-aligned and new phylogenies reconstructed. OsAMT1-2 was the only functionally characterized sequence in this category and was excluded from the phylogeny. Bootstrap values were reported within groups (as color coded stars for the three methods) if the support was higher than 75%. Maximum Likelihood bootstrap values were reported on all branches outside groups and were supplemented with colored stars, when the same topology was also supported by distance and/or parsimony analyses. To gather evidence for monophyly of the families within plants, sequences from each group of each family were used as probes in additional BLAST searches against the GenBank database (excluding *Viridiplantae* sequences) to identify publically available non-plant sequences sharing sequence similarity with the baits. The ten top BLAST hits (lowest expect value) were retained. These sequences were added to the respective sequence collection, the family was re-aligned and new phylogenies were reconstructed. If the non-*Viridiplantae* sequences formed a separate clan, the plant family was considered monophyletic.

### Expression profiling, transmembrane predictions, subcellular predictions

Subcellular localization predictions were performed using MultiLoc2 [79] with the MultiLoc2-HighRes (Plant), 10 Locations algorithm. All predictions were recorded, but only the highest probability prediction was reported in the final figures. TM domain predictions were performed using TopCons [80] with no restrainment options selected. The TopCons website reports on several topology prediction programs’ results in addition to the TopCons-exclusive prediction, but only the TopCons-exclusive prediction was recorded here. *In silico* expression profiling (heatmapping) was performed using the Bio-Array for Plant Biology (BAR) eFP (electronic fluorescent pictograph) browser, which is based on re-normalized Affymetrix® microarray expression data published previously [81,82]. Tissue and organ gene expression data for each gene were retrieved from the respective eFP browser site and compiled into a data table. This was used to generate heatmaps where colour coding was used to visualize expression levels. These visualizations were performed using Microsoft Excel. Organisms analyzed include *A. thaliana*, *P. trichocarpa*, and *O. sativa*.

### Supporting data

The data sets supporting the results of this article are included within the article and its additional files. Accession numbers and sequences of proteins included are given in Additional file 1. The alignment and tree files presented have been submitted to TreeBASE (accession number 14948).

## Endnote

^a^While this manuscript was under review an alternative naming and classification system of the NRT1/PTR superfamily was proposed by Léran et al. (Trends Plant Sci, in press, doi:10.1016/j.tplants.2013.08.008). Largely, the ‘supergroups’ described here and the ‘clades’ defined by Léran et al. have good correspondence, albeit relationships between ‘supergroups/clades’ lack resolution and thus correspondence: supergroup A corresponds to clade 4; supergroups B, E, and J together correspond to clade 6, supergroup C corresponds to clade 3; supergroup D corresponds to clades 1 and 2; supergroup F corresponds largely to clade 8, but the distinct group F II-M (defined by OsPTR6 in Figure 1) was placed into clade 7; the remainder of clade 7 corresponds to supergroup G; supergroups H and I together correspond to clade 5. For ease of comparison, the names used by Léran et al. were added to Additional file 1.

## Abbreviations

N: Nitrogen; AMT: Ammonium transporter; NRT: Nitrate transporter; PTR: Peptide transporter; HATS: High affinity transport system; LATS: Low affinity transport system.

## Competing interests

The authors declare that they have no competing interests.

## Authors’ contributions

JE designed the study, performed analyses, and drafted the paper. NJJBvW performed analyses and drafted the paper. CHL performed analyses and interpreted results. BJH designed the study and drafted the paper. All authors read and approved the final manuscript.

## Supplementary Material

Additional file 1 Protein sequences included in phylogenetic analyses including name used, categorization applied, species of origin, database accessions (GenBank for non-plant sequences, Phytozome v6.0 for plant sequences), and protein sequence. Click here for file

Additional file 2 **Maximum Likelihood phylogenetic reconstructions of the NRT1 family by supergroup.** For each tree, sequences were realigned and trimmed separately prior to phylogenetic reconstruction. Groups are defined and colored as in Figure 1. Bootstrap values from 1,000 replicates are given for branches up to those defining groups only. Supergroups C and D, supergroups F and G, and supergroups H and I were analyzed together, because they each form monophyletic clans with high bootstrap support (see Figure 1) but have bryophyte and lycophyte proteins with ambiguous relation to the two supergroups included. Click here for file
