# Supplementary material for: Evolutionary classification of ammonium, nitrate, and peptide transporters in land plants
Source: BMC Evol Biol. 2014 Jan 20;14:11. doi: 10.1186/1471-2148-14-11 (PMC3922906; doi:10.1186/1471-2148-14-11)

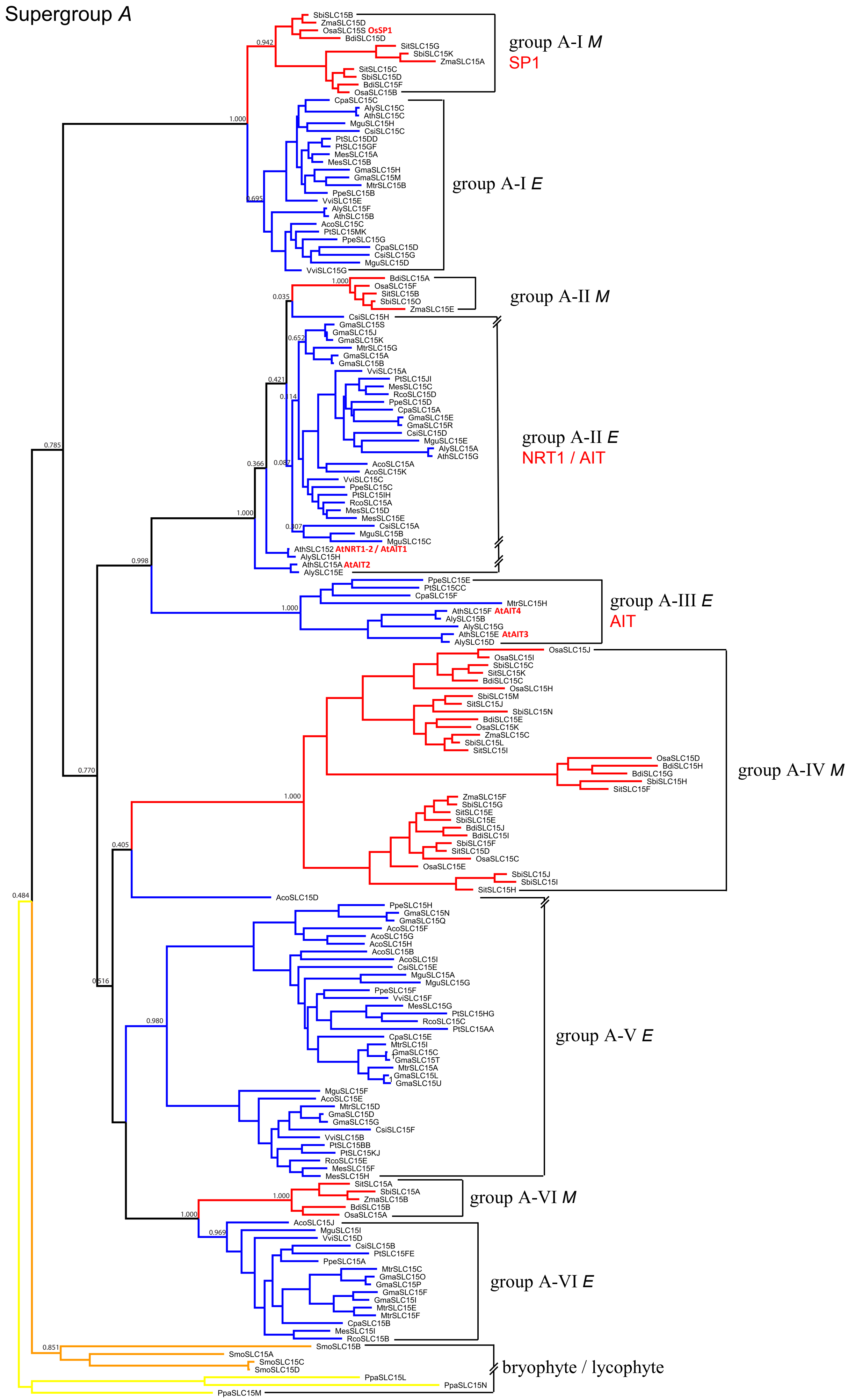
Supergroup A
SbiSLC15B
ZmaSLC15D
OsaSLC15S OsSP1
BdiSLC15D
SitSLC15G
SbiSLC15K
ZmaSLC15A
SitSLC15C
SbiSLC15D
BdiSLC15F
OsaSLC15B
group A-I M
SP1
CpaSLC15C
AlySLC15C
AthSLC15C
MguSLC15H
CsiSLC15C
PtSLC15DD
PtSLC15GF
MesSLC15A
MesSLC15B
GmaSLC15H
GmaSLC15M
MtrSLC15B
PpeSLC15B
VviSLC15E
AlySLC15F
AthSLC15B
AcoSLC15C
PtSLC15MK
PpeSLC15G
CpaSLC15D
CsiSLC15G
MguSLC15D
VviSLC15G
group A-I E
BdiSLC15A
OsaSLC15F
SitSLC15B
SbiSLC15O
ZmaSLC15E
group A-II M
CsiSLC15H
GmaSLC15S
GmaSLC15J
GmaSLC15K
MtrSLC15G
GmaSLC15A
GmaSLC15B
VviSLC15A
PtSLC15JI
MesSLC15C
RcoSLC15D
PpeSLC15D
CpaSLC15A
GmaSLC15E
GmaSLC15R
CsiSLC15D
MguSLC15E
AlySLC15A
AthSLC15G
AcoSLC15A
AcoSLC15K
VviSLC15C
PpeSLC15C
PtSLC15IH
RcoSLC15A
MesSLC15D
MesSLC15E
CsiSLC15A
MguSLC15B
MguSLC15C
AthSLC152 AtNRT1-2 / AtAIT1
AlySLC15H
AthSLC15A AtAIT2
AlySLC15E
group A-II E
NRT1 / AIT
PpeSLC15E
PtSLC15CC
CpaSLC15F
MtrSLC15H
AthSLC15F AtAIT4
AlySLC15B
AlySLC15G
AthSLC15E AtAIT3
AlySLC15D
group A-III E
AIT
OsaSLC15J
OsaSLC15I
SbiSLC15C
SitSLC15K
BdiSLC15C
OsaSLC15H
SbiSLC15M
SitSLC15J
SbiSLC15N
BdiSLC15E
OsaSLC15K
ZmaSLC15C
SbiSLC15L
SitSLC15I
OsaSLC15D
BdiSLC15H
BdiSLC15G
SbiSLC15H
SitSLC15F
ZmaSLC15F
SbiSLC15G
SitSLC15E
SitSLC15E
BdiSLC15J
BdiSLC15I
SbiSLC15F
SitSLC15D
OsaSLC15C
OsaSLC15E
SbiSLC15J
SbiSLC15I
SitSLC15H
group A-IV M
AcoSLC15D
PpeSLC15H
GmaSLC15N
GmaSLC15Q
AcoSLC15F
AcoSLC15G
AcoSLC15H
AcoSLC15B
AcoSLC15I
CsiSLC15E
MguSLC15A
MguSLC15G
PpeSLC15F
VviSLC15F
MesSLC15G
PtSLC15HG
RcoSLC15C
PtSLC15AA
CpaSLC15E
MtrSLC15I
GmaSLC15C
GmaSLC15T
MtrSLC15A
GmaSLC15L
GmaSLC15U
MguSLC15F
AcoSLC15E
MtrSLC15D
GmaSLC15D
GmaSLC15G
CsiSLC15F
VviSLC15B
PtSLC15BB
PtSLC15KJ
RcoSLC15E
MesSLC15F
MesSLC15H
group A-V E
SitSLC15A
SbiSLC15A
ZmaSLC15B
BdiSLC15B
OsaSLC15A
group A-VI M
AcoSLC15J
MguSLC15I
VviSLC15D
CsiSLC15B
PtSLC15FE
PpeSLC15A
MtrSLC15C
GmaSLC15O
GmaSLC15P
GmaSLC15F
GmaSLC15I
MtrSLC15E
MtrSLC15F
CpaSLC15B
MesSLC15I
RcoSLC15B
group A-VI E
SmoSLC15B
SmoSLC15A
SmoSLC15C
SmoSLC15D
PpaSLC15L
PpaSLC15N
PpaSLC15M
bryophyte / lycophyte
0.942
1.000
0.695
1.000
0.035
0.652
0.421
0.114
0.366
0.087
1.000
0.207
0.998
1.000
0.785
0.770
1.000
0.405
0.516
0.980
1.000
1.000
0.969
0.484
0.851

Supergroup *B*

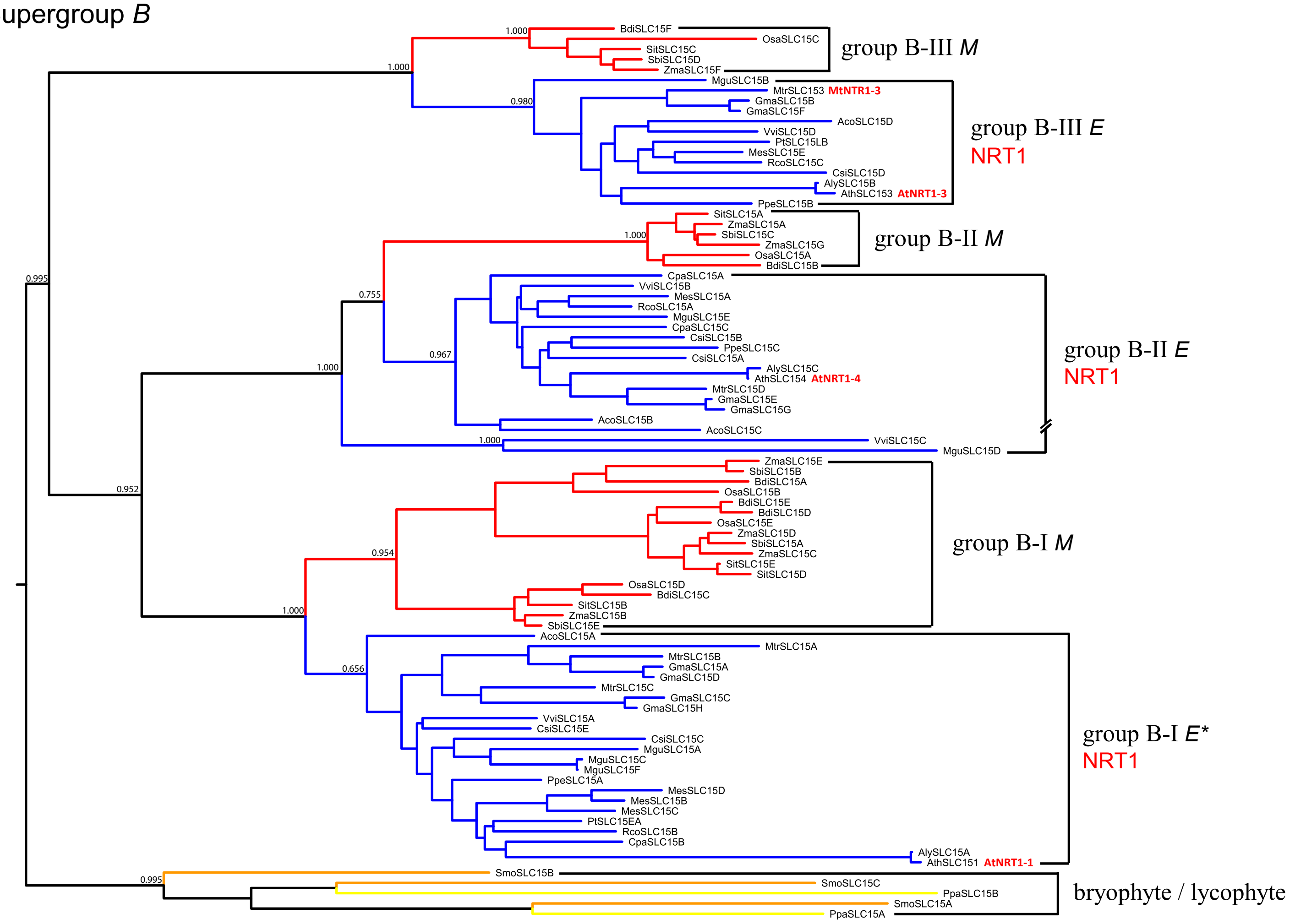

Supergroup *C*

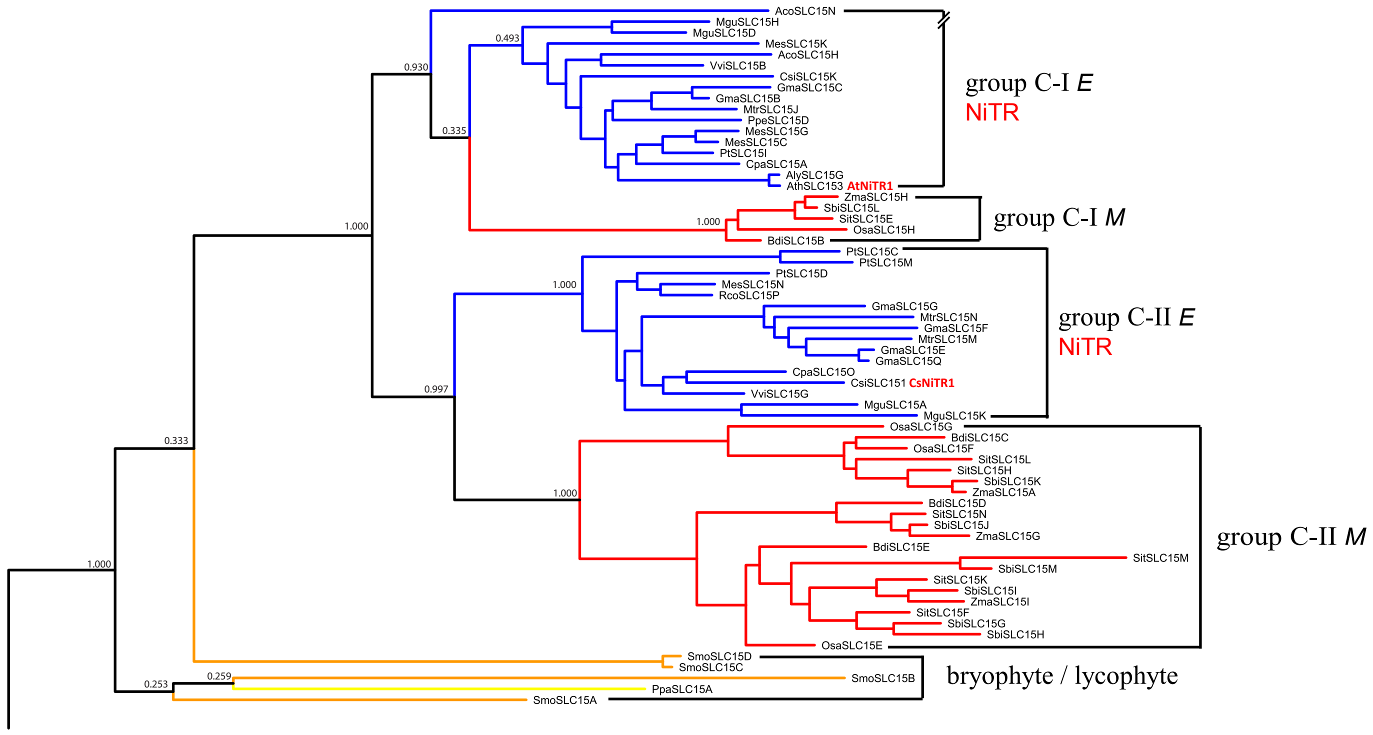

tree continued
on next page
(supergroup D)

Supergroup *D*

tree continued from last page (supergroup C)

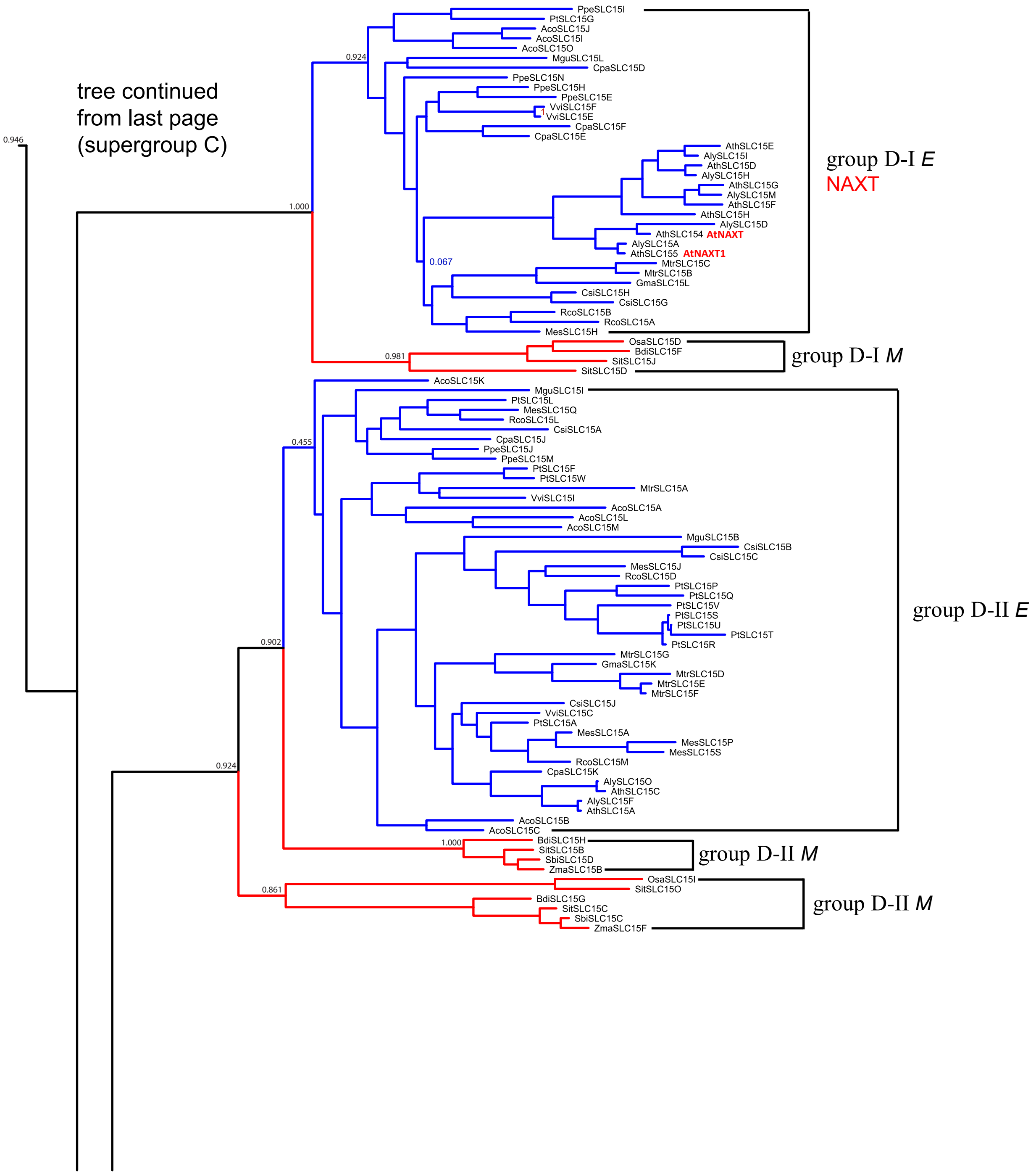

supergroup D continued on next page

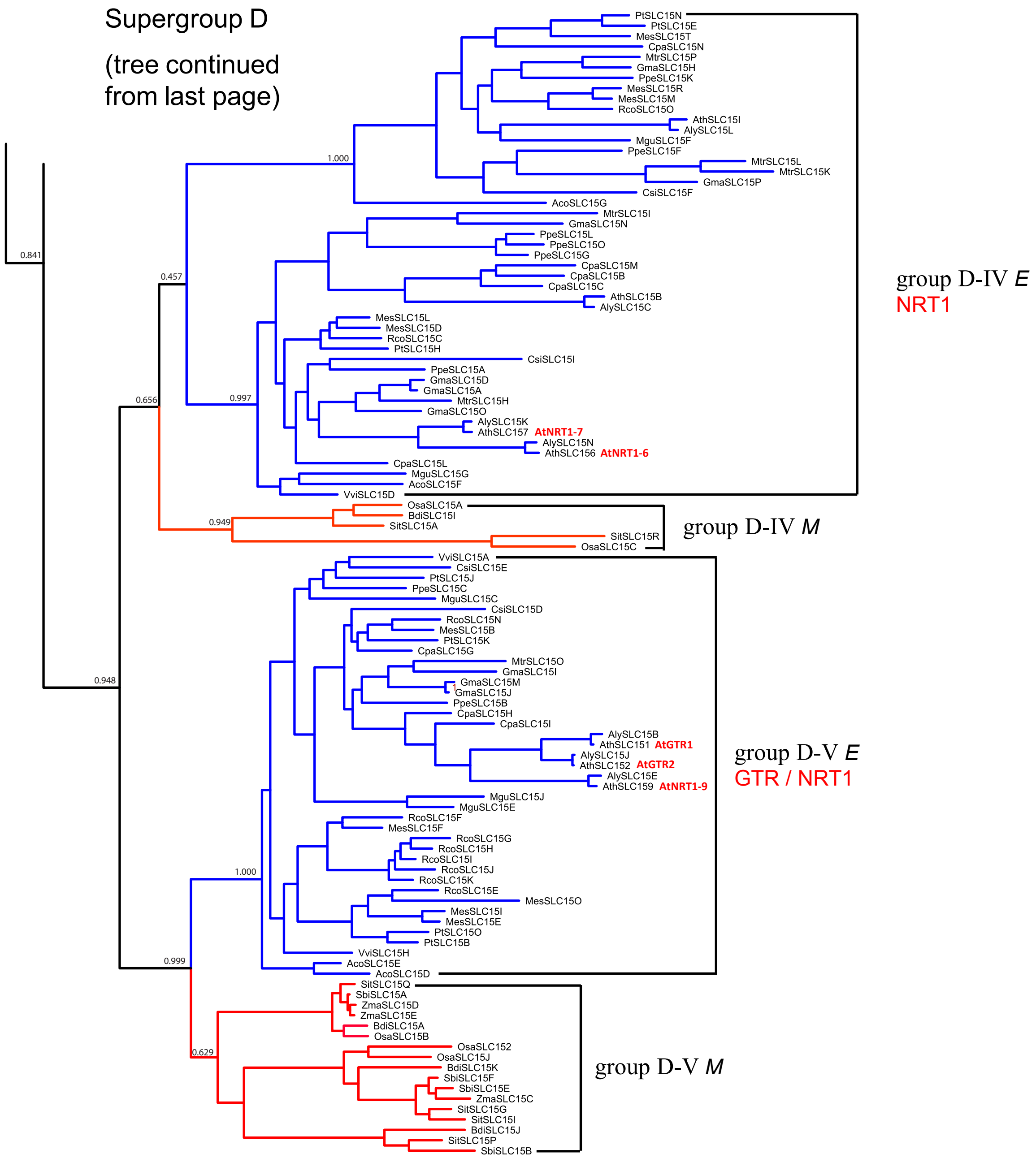

Supergroup D
(tree continued from last page)
PtSLC15N
PtSLC15E
MesSLC15T
CpaSLC15N
MtrSLC15P
GmaSLC15H
PpeSLC15K
MesSLC15R
MesSLC15M
RcoSLC15O
AthSLC15I
AlySLC15L
MguSLC15F
PpeSLC15F
MtrSLC15L
MtrSLC15K
GmaSLC15P
CsiSLC15F
AcoSLC15G
MtrSLC15I
GmaSLC15N
PpeSLC15L
PpeSLC15O
PpeSLC15G
CpaSLC15M
CpaSLC15B
CpaSLC15C
AthSLC15B
AlySLC15C
MesSLC15L
MesSLC15D
RcoSLC15C
PtSLC15H
CsiSLC15I
PpeSLC15A
GmaSLC15D
GmaSLC15A
MtrSLC15H
GmaSLC15O
AlySLC15K
AthSLC157 AtNRT1-7
AlySLC15N
AthSLC156 AtNRT1-6
CpaSLC15L
MguSLC15G
AcoSLC15F
VviSLC15D
OsaSLC15A
BdiSLC15I
SitSLC15A
SitSLC15R
OsaSLC15C
VviSLC15A
CsiSLC15E
PtSLC15J
PpeSLC15C
MguSLC15C
CsiSLC15D
RcoSLC15N
MesSLC15B
PtSLC15K
CpaSLC15G
MtrSLC15O
GmaSLC15I
GmaSLC15M
GmaSLC15J
PpeSLC15B
CpaSLC15H
CpaSLC15I
AlySLC15B
AthSLC151 AtGTR1
AlySLC15J
AthSLC152 AtGTR2
AlySLC15E
AthSLC159 AtNRT1-9
MguSLC15J
MguSLC15E
RcoSLC15F
MesSLC15F
RcoSLC15G
RcoSLC15H
RcoSLC15I
RcoSLC15J
RcoSLC15K
RcoSLC15E
MesSLC15O
MesSLC15I
MesSLC15E
PtSLC15O
PtSLC15B
VviSLC15H
AcoSLC15E
AcoSLC15D
SitSLC15Q
SbiSLC15A
ZmaSLC15D
ZmaSLC15E
BdiSLC15A
OsaSLC15B
OsaSLC152
OsaSLC15J
BdiSLC15K
SbiSLC15F
SbiSLC15E
ZmaSLC15C
SitSLC15G
SitSLC15I
BdiSLC15J
SitSLC15P
SbiSLC15B
1.000
0.841
0.457
0.656
0.997
0.949
0.948
1.000
0.999
0.629
group D-IV E
NRT1
group D-IV M
group D-V E
GTR / NRT1
group D-V M

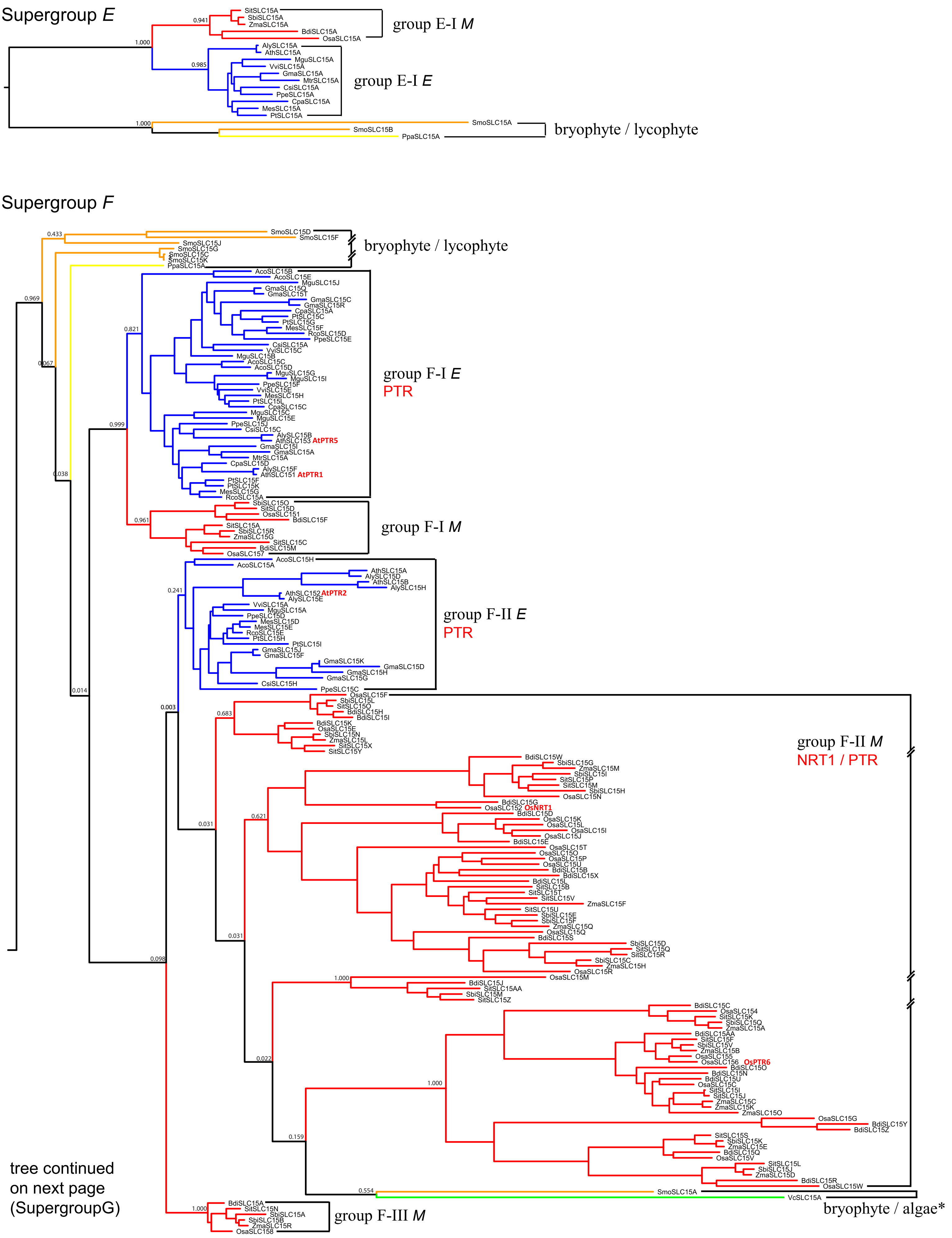

tree continued on next page (SupergroupG)

Supergroup G

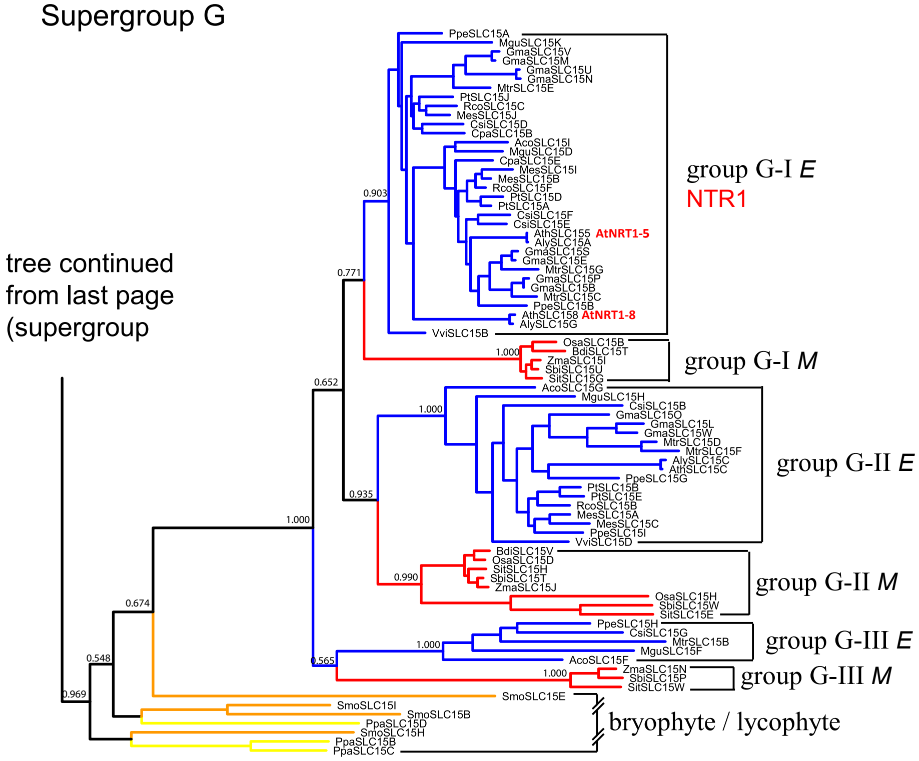

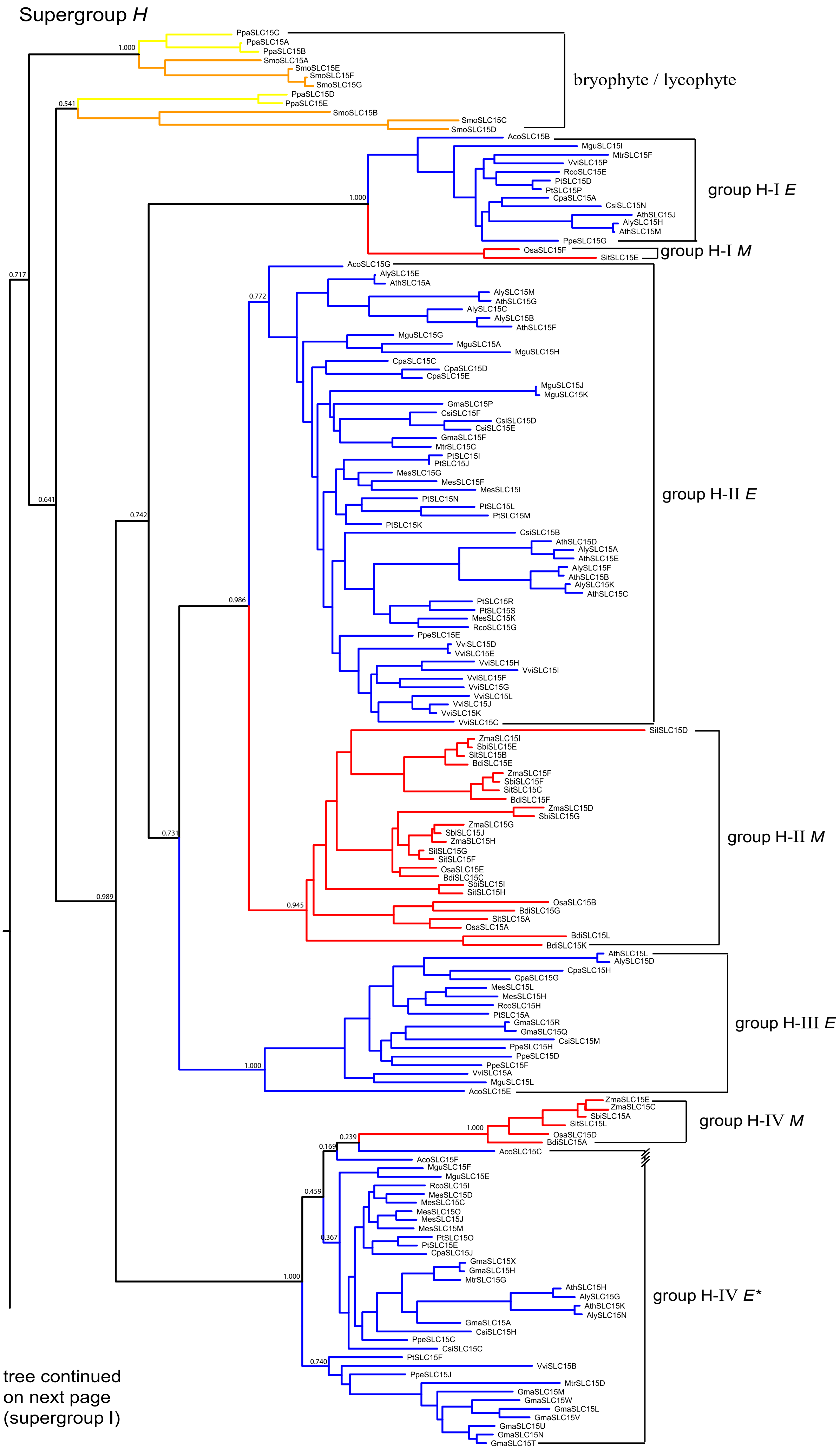
Supergroup H
PpaSLC15C
PpaSLC15A
PpaSLC15B
SmoSLC15A
SmoSLC15E
SmoSLC15F
SmoSLC15G
PpaSLC15D
PpaSLC15E
SmoSLC15B
SmoSLC15C
SmoSLC15D
bryophyte / lycophyte
AcoSLC15B
MguSLC15I
MtrSLC15F
VviSLC15P
RcoSLC15E
PtSLC15D
PtSLC15P
CpaSLC15A
CsiSLC15N
AthSLC15J
AlySLC15H
AthSLC15M
PpeSLC15G
group H-I E
OsaSLC15F
SitSLC15E
group H-I M
AcoSLC15G
AlySLC15E
AthSLC15A
AlySLC15M
AthSLC15G
AlySLC15C
AlySLC15B
AthSLC15F
MguSLC15G
MguSLC15A
MguSLC15H
CpaSLC15C
CpaSLC15D
CpaSLC15E
MguSLC15J
MguSLC15K
GmaSLC15P
CsiSLC15F
CsiSLC15D
CsiSLC15E
GmaSLC15F
MtrSLC15C
PtSLC15I
PtSLC15J
MesSLC15G
MesSLC15F
MesSLC15I
PtSLC15N
PtSLC15L
PtSLC15M
PtSLC15K
CsiSLC15B
AthSLC15D
AlySLC15A
AthSLC15E
AlySLC15F
AthSLC15B
AlySLC15K
AthSLC15C
PtSLC15R
PtSLC15S
MesSLC15K
RcoSLC15G
PpeSLC15E
VviSLC15D
VviSLC15E
VviSLC15H
VviSLC15I
VviSLC15F
VviSLC15G
VviSLC15L
VviSLC15J
VviSLC15K
VviSLC15C
group H-II E
SitSLC15D
ZmaSLC15I
SbiSLC15E
SitSLC15B
BdiSLC15E
ZmaSLC15F
SbiSLC15F
SitSLC15C
BdiSLC15F
ZmaSLC15D
SbiSLC15G
ZmaSLC15G
SbiSLC15J
ZmaSLC15H
SitSLC15G
SitSLC15F
OsaSLC15E
BdiSLC15C
SbiSLC15I
SitSLC15H
OsaSLC15B
BdiSLC15G
SitSLC15A
OsaSLC15A
BdiSLC15L
BdiSLC15K
group H-II M
AthSLC15L
AlySLC15D
CpaSLC15H
CpaSLC15G
MesSLC15L
MesSLC15H
RcoSLC15H
PtSLC15A
GmaSLC15R
GmaSLC15Q
CsiSLC15M
PpeSLC15H
PpeSLC15D
PpeSLC15F
VviSLC15A
MguSLC15L
AcoSLC15E
group H-III E
ZmaSLC15E
ZmaSLC15C
SbiSLC15A
SitSLC15L
OsaSLC15D
BdiSLC15A
group H-IV M
AcoSLC15C
AcoSLC15F
MguSLC15F
MguSLC15E
RcoSLC15I
MesSLC15D
MesSLC15C
MesSLC15O
MesSLC15J
MesSLC15M
PtSLC15O
PtSLC15E
CpaSLC15J
GmaSLC15X
GmaSLC15H
MtrSLC15G
AthSLC15H
AlySLC15G
AthSLC15K
AlySLC15N
GmaSLC15A
CsiSLC15H
PpeSLC15C
CsiSLC15C
PtSLC15F
VviSLC15B
PpeSLC15J
MtrSLC15D
GmaSLC15M
GmaSLC15W
GmaSLC15L
GmaSLC15V
GmaSLC15U
GmaSLC15N
GmaSLC15T
group H-IV E*
1.000
0.541
0.717
0.641
1.000
0.742
0.772
0.986
0.731
0.989
0.945
1.000
0.239
0.169
1.000
0.459
0.367
1.000
0.740
tree continued on next page (supergroup I)

Supergroup *I*

tree continued
from last page
(supergroup H)

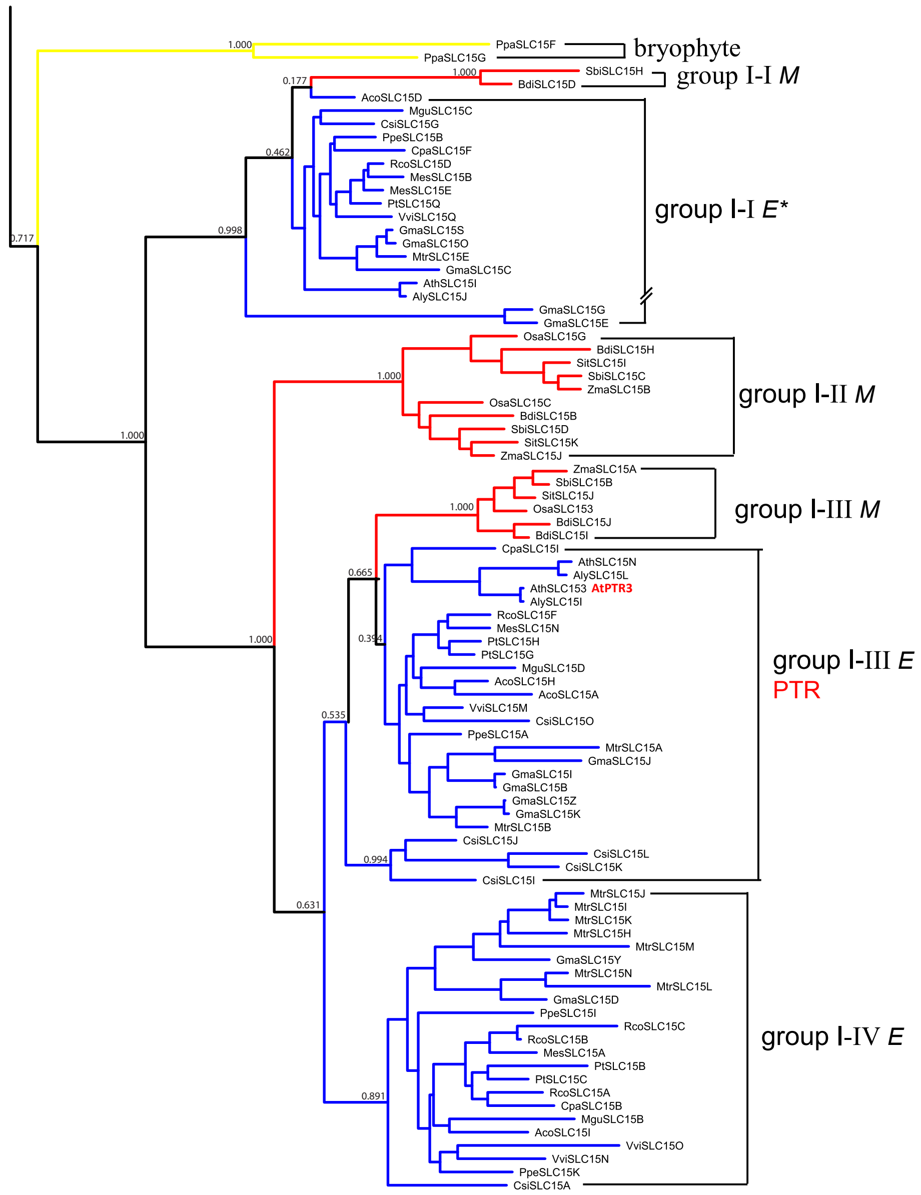

Supergroup J

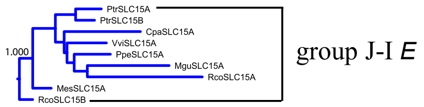

Supplement: Additional file 2 — Maximum Likelihood phylogenetic reconstructions of the NRT1 family by supergroup. For each tree, sequences were realigned and trimmed separately prior to phylogenetic reconstruction. Groups are defined and colored as in Figure 1. Bootstrap values from 1,000 replicates are given for branches up to those defining groups only. Supergroups C and D, supergroups F and G, and supergroups H and I were analyzed together, because they each form monophyletic clans with high bootstrap support (see Figure 1) but have bryophyte and lycophyte proteins with ambiguous relation to the two supergroups included. [file 1471-2148-14-11-S2.pdf]
